# Construction and validation of a prognostic model of RNA binding proteins in clear cell renal carcinoma

**DOI:** 10.1186/s12882-022-02801-y

**Published:** 2022-05-05

**Authors:** Wenkai Han, Bohao Fan, Yongshen Huang, Xiongbao Wang, Zhao Zhang, Gangli Gu, Zhao Liu

**Affiliations:** 1 Department of Clinical Medicine, Qingdao University, Qingdao, Shandong 266000 China; 2 Department of Urology, Qilu Hospital of Shandong University, Jinan, Shandong 250012 China

**Keywords:** Clear cell renal carcinoma, RNA binding proteins, Risk model

## Abstract

**Background:**

The dysfunction of RNA binding proteins (RBPs) is associated with various inflammation and cancer. The occurrence and progression of tumors are closely related to the abnormal expression of RBPs. There are few studies on RBPs in clear cell renal carcinoma (ccRCC), which allows us to explore the role of RBPs in ccRCC.

**Methods:**

We obtained the gene expression data and clinical data of ccRCC from the Cancer Genome Atlas (TCGA) database and extracted all the information of RBPs. We performed differential expression analysis of RBPs. Risk model were constructed based on the differentially expressed RBPs (DERBPs). The expression levels of model markers were examined by reverse transcription-quantitative PCR (RT-qPCR) and analyzed for model-clinical relevance. Finally, we mapped the model's nomograms to predict the 1, 3 and 5-year survival rates for ccRCC patients.

**Results:**

The results showed that the five-year survival rate for the high-risk group was 40.2% (95% CI = 0.313 ~ 0.518), while the five-year survival rate for the low-risk group was 84.3% (95% CI = 0.767 ~ 0.926). The ROC curves (AUC = 0.748) also showed that our model had stable predictive power. Further RT-qPCR results were in accordance with our analysis (*p* < 0.05). The results of the independent prognostic analysis showed that the model could be an independent prognostic factor for ccRCC. The results of the correlation analysis also demonstrated the good predictive ability of the model.

**Conclusion:**

In summary, the 4-RBPs (EZH2, RPL22L1, RNASE2, U2AF1L4) risk model could be used as a prognostic indicator of ccRCC. Our study provides a possibility for predicting the survival of ccRCC.

**Supplementary Information:**

The online version contains supplementary material available at 10.1186/s12882-022-02801-y.

## Background

Among urological tumors, renal cell carcinoma (RCC) is one of the most prevalent malignancies [1]. The most common subtype of RCC is clear cell renal cell carcinoma (ccRCC), which accounts for about 4/5 of all renal cell carcinomas [2, 3]. With the increase in early diagnosis of ccRCC, the survival rate for stage 1 and 2 renal cell carcinoma (RCC) is above 90% [4]. However, there are limitations in the treatment of advanced ccRCC and the prognosis for the patients is poor, with a 5-year survival rate of less than 10%, and about 1/3 of patients develop metastatic disease after treatment [5]. Although there has been significant progress in the treatment of late stage ccRCC, such as tyrosine-kinase inhibitors (TKIs) and immune checkpoint therapies (ICTs), most patients were refractory due to tumor heterogeneity and lack of effective signature predicting efficacy and prognosis [6]. Therefore, it is urgently needed to develop new markers to improve the individual therapy and prognosis of ccRCC.

RNA binding proteins (RBPs) can interact with various types of RNA and play their biological functions. At present, 1,542 RBP related genes have been identified in the human genome [7]. RBPs play roles in maintaining the physiological balance of cells and are particularly important in the development process and stress response [8]. In RNA metabolism, RBPs are involved in selective splicing, modification, localization and translation [9, 10]. It has been proved that RBPs have an essential relationship with the occurrence of many diseases [11]. Although RBPs control transcriptional metabolites to influence tumor development and progression, the role of RBPs in tumor remains unclear [12]. Studies have shown that RBPs are differentially expressed between normal and tumor tissues, affecting the translation process of mRNA and occurrence of tumors [13, 14]. In addition, a series of studies have reported that differential expression of RBP related with prognosis in different cancer patients [15, 16]. To date, there has been a relative lack of research on RBPs in ccRCC.

In this study, we investigated the role of RBPs in ccRCC and its correlation with patient survival. The raw data of ccRCC was obtained from the TCGA database. The differential expressed RBPs (DERBPs) between tumor tissues and normal tissues were analyzed, and the biological function analysis and protein–protein interaction analysis of DERBPs were performed. Risk prediction model were constructed based on DERBPs as a way to predict patient prognosis. The expression levels of model markers were examined in cancerous tissue, normal renal tissue, renal cancer cell lines and non-renal cancer cell lines using RT-qPCR. Finally, we explored the clinical relevance of risk marker genes and constructed a nomogram to formulate precise clinical prognosis strategies effectively.

## Materials and methods

### Cell culture

Human Embryonic Renal Cell Line (293 T), Renal non-cancer cell line (HK2), Human renal carcinoma cell lines 786-O and OS-RC-2 were obtained from the Typical Culture Preservation Commission Cell Bank, Chinese Academy of Sciences (Shanghai, China). Materials for the cell culture process, including Fetal Bovine Serum (FBS), RPMI 1640 culture medium, trypsin, penicillin and streptomycin, were purchased from Gibco (Grand Island, NY, USA). The culture medium for all cell lines contained 90% RPMI 1640, 10% FBS and 1% antibiotics (100 U/ml streptomycins and 100 U/ml penicillin). All cell lines were cultured at 37 °C, 5% CO_2_.

Each cell was inoculated at a density of 1 × 10^6^ cells/well in 6-well plates, 2 ml of mixed media per well and incubated for 24 h at 37 °C in a humidified incubator with 5% CO_2_. Total RNA was extracted from the 6-well plates using Trizol reagent (Vazyme, China).

### Patients and samples

Twenty pairs of clear cell renal cancer tissues and paraneoplastic tissue specimens were obtained from patients after radical surgery for clear cell renal cancer at the Affiliated Hospital of Medical College of Qingdao University. The detailed clinicopathological characteristics of the 20 ccRCC patients are shown in Table 1. The cancerous and paraneoplastic tissues (5 cm apart) were rinsed in sterile PBS and rapidly frozen in liquid nitrogen within 30 min after removal. The Institutional Review Board approved the study protocol, and informed consent was obtained from the patients.

**Table 1:** Our ccRCC patients’ characteristics. The proportion of each clinical characteristics in our sample (Grade: G1, highly differentiated; G2, moderately differentiated; G3, poorly differentiated; G4, undifferentiated; T status: T1, Tumour confined to the renal with a maximum diameter ≤ 7 cm; T2, Tumour confined to the renal with a maximum diameter > 7 cm; T3, Tumour invading a segmental or renal vein or the inferior vena cava, or invading perirenal tissue without invading the ipsilateral adrenal gland or exceeding the perirenal fascia; T4, Tumour invading the perirenal fascia, including invading the ipsilateral adrenal gland. M status: M0, No distant metastases; M1 with distant metastases. N status: N0, No regional lymph node metastases; N1, With regional lymph node metastase.)

Clinical characteristics	Total (*n* = 20)	Percent (%)
Age		
<60	5	25
≥ 60	15	75
Gender		
Female	9	45
Male	11	55
Grade		
G1	1	5
G2	4	20
G3	11	55
G4	4	20
Stage		
Stage I	5	25
Stage II	2	10
Stage III	6	30
Stage IV	7	35
T		
T1	5	25
T2	3	15
T3	9	45
T4	3	15
N		
N0	14	70
N1	6	30
M		
M0	13	65
M1	7	35

### Data preparation

**Fig. 1:**
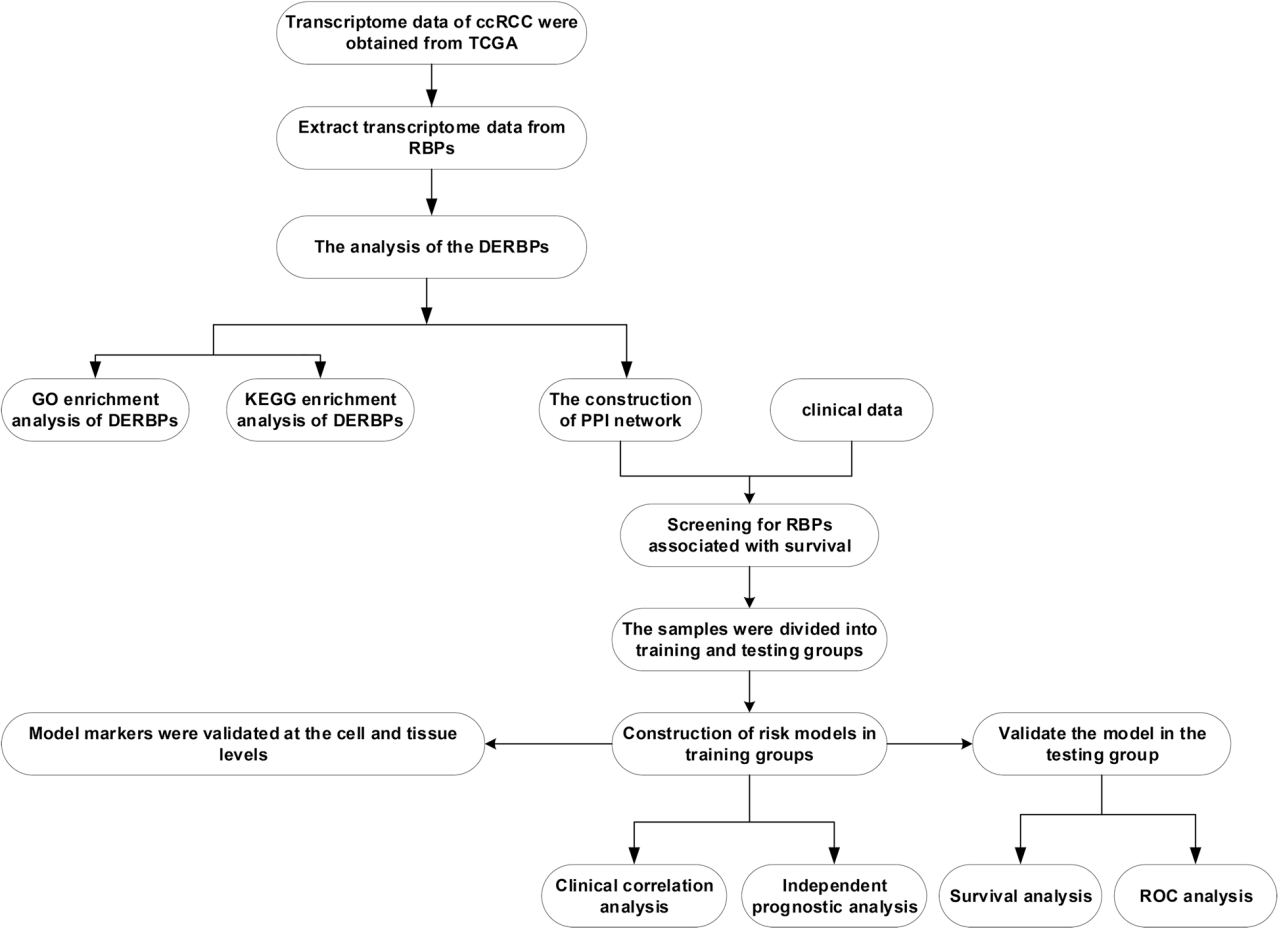
The flow chart of this study

We obtained all transcriptome (FPKM) and clinical data of ccRCC from the TCGA (https://portal.gdc.cancer.gov/) database, it contains 72 paraneoplastic samples and 539 tumor samples. In human cells, 1542 RBPs genes (Supplement Table 1) were screened by high-throughput screening [7]. We extracted 1495 RBPs genes expression data from TCGA data. The process of this study follows the flow chart (Fig. 1).

### Differential expression analysis of RBPs genes

To observe whether RBPs genes are expressed differently in tumor and normal tissues. In R (Version 3.6.2) language environment, “limma" packages (http://www.bioconductor.org/packages/release/bioc/html/limma.html) were used for data correction, and Wilcox test was used for data difference analysis. The cut-off value of our screening criteria was |logFC|> 1 and FDR < 0.05 (Adjusted *p*-value). Heat maps were drawn using “pheatmap” package, and volcanoes were mapped. Finally, we collate the differential expression RBPs (DERBPs) output into a readable file.

### Biological functional analysis of DERBPs

To further understand the biological function of DERBPs in ccRCC. We carried out Gene Ontology (GO) [17] enrichment analysis and Kyoto Encyclopedia of Genes and Genomes (KEGG) [18] pathway enrichment analysis of DERBPs. A *p*-value < 0.05 and FDR < 0.05 was used as the GO and KEGG enrichment analysis filtration standard. The analysis process is carried out in the R language environment. The R packages used include "clusterProfiler", "ggplot2" and" enrichplot".

### Construction of protein–protein interaction (PPI) network of DERBPs

To explore the interaction between DERBPs. We used STRING (http://www.string-db.org/, Version 11.0), an online analysis tool, for PPI analysis [19]. Visualise the PPI network using Cytoscape (Version 3.7.2).

### Construction of the RBPs prognostic risk model

To further verify the model's prediction efficiency, samples were randomly divided into training group and test group in a 1:1 ratio. We obtained information on a total of 537 patients from the TCGA database. A total of 530 patient sample data were obtained by excluding samples with missing data. The training group contained 266 samples, while the test group contained 264 samples. We screened the prognosis genes associated with ccRCC survival from the PPI network. We performed univariate Cox regression analysis of genes in the network to identify genes associated with overall survival (*p* < 0.0001). Finally, we performed multivariate Cox regression analysis of genes related to survival, constructed a risk prediction model of RBPs, and calculated the patients' risk scores in the training group. Risk score = Ʃ (β_n_ × Exp_n_). In the formula, β represents the regression coefficient, and Exp represents the expression level of related genes. All operations for this step are performed in the R language environment. The R package used in the operation procedure includes "survival", "caret", "glmnet" and "survminer".

### Validation of the RBPs risk model

We divided the 264 samples in the test group into high-risk and low-risk groups based on the median risk score of the model. The overall survival of the two groups was observed. We also draw the receiver operating characteristic (ROC) curve to evaluate our prediction effectiveness and by using the "survivalROC” package in R.

### Risk model and clinical correlation analysis

To further investigate the relevance of the model to clinical practice. In the R environment, the chi-square test is used for analysis. We were able to assess the correlation between the model and clinicopathological features. We also performed an independent predictive analysis of the model to determine whether our model can be used as a prognostic indicator of ccRCC alone. To better apply the clinical practice model, we drew a nomogram to predict patients' survival status accurately.

### Reverse transcription‑quantitative PCR analysis

To further analyze the accuracy of the model, we validated the expression levels of model-related genes at the tissue and cell line levels, respectively. The procedure for tissue extraction of RNA is provided in Supplementary Material (S1). For cells, total RNA was extracted from the 6-well plates using Trizol reagent (Vazyme, China). A microgram of total RNA was reverse transcribed to cDNA with HiScript III RT SuperMix for qPCR Reverse Transcriptase (Vazyme, China). Quantitative real-time PCR was performed to analyse the cDNA according to the SYBR Color qPCR Master Mix (Vazyme, China) instructions with a Roche LightCycler 480II real-time PCR detection system (Roche, Switzerland). Normalization was carried out using GAPDH. Details of the primers used in this experiment are given in Table 2.

**Table 2:**
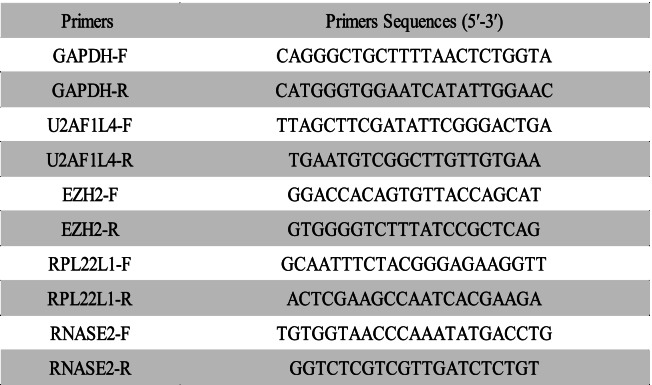
All sequences used in this study

### Statistical analysis

The data mining and processing in this study were done using R software (version 3.6.2). All the experimental data (PCR data) in this study have been repeated 3 times and are expressed as $\overline{\mathrm X}$ ± S. The experimental data (PCR data) were all statistically analyzed using SPSS software (version 26.0) and the data were visualised using Graphpad Prism software. For the subgroups in this experiment where only two groups were compared, we used the t-test for comparison, and for data containing more than two subgroups, we used one-way ANOVA for statistical analysis. The analysis results *p* < 0.05 considered the differences to be statistically significant.

## Results

### Differential expression analysis of RBPs

**Fig. 2:**
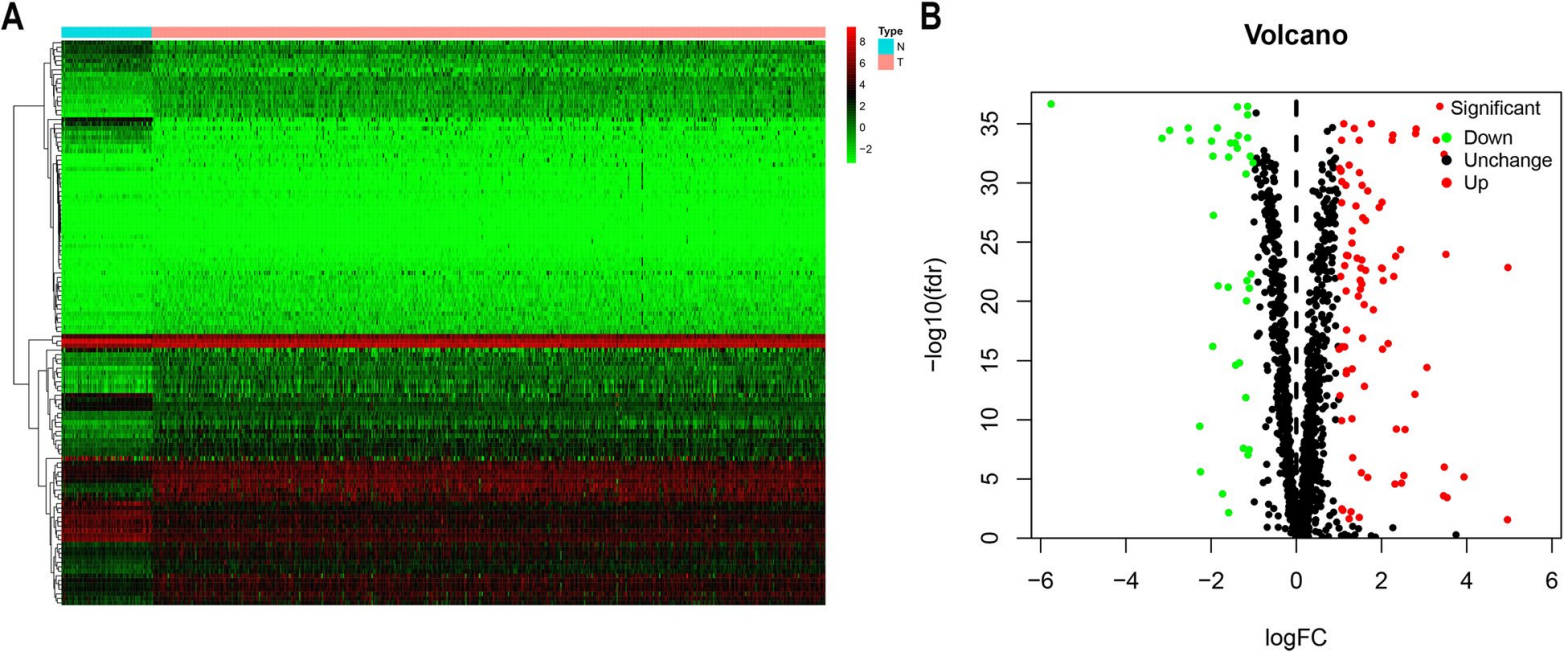
Differential expression RBPs (DERBPs) of TCGA ccRCC data. The RNA expression matrix of 72 paracancer samples and 539 tumour samples were analysed for differential expression, statistically using the wilcoxon test, with a cut-off of |logFC|> 1 and FDR < 0.05 (Adjusted *p*-value). **A** Differential expression heatmap. Each vertical column represents each sample, and each row represents an RBP gene. **B** Differential expression volcanic map. The volcano gram shows the differential expression of DERBPs, with green indicating down-regulated, red indicating up-regulated, and black indicating no difference. N, normal; T, tumor

In R language environment, 1495 RBPs-related genes in ccRCC were analyzed according to the screening criteria (|log FC|> 1, FDR < 0.05). The RNA expression matrix of 72 paracancer samples and 539 tumor samples were analysed for differential expression, statistically using the wilcoxon test. The results showed 125 dysregulated RBPs in tumor tissues, among which 38 genes were up-regulated, and 87 were down-regulated (Supplement Table 2). We visualized the differentially expressed genes by mapping heat map and volcanic maps (Fig. 2).

### GO and KEGG enrichment analysis of DERBPs

**Fig. 3:**
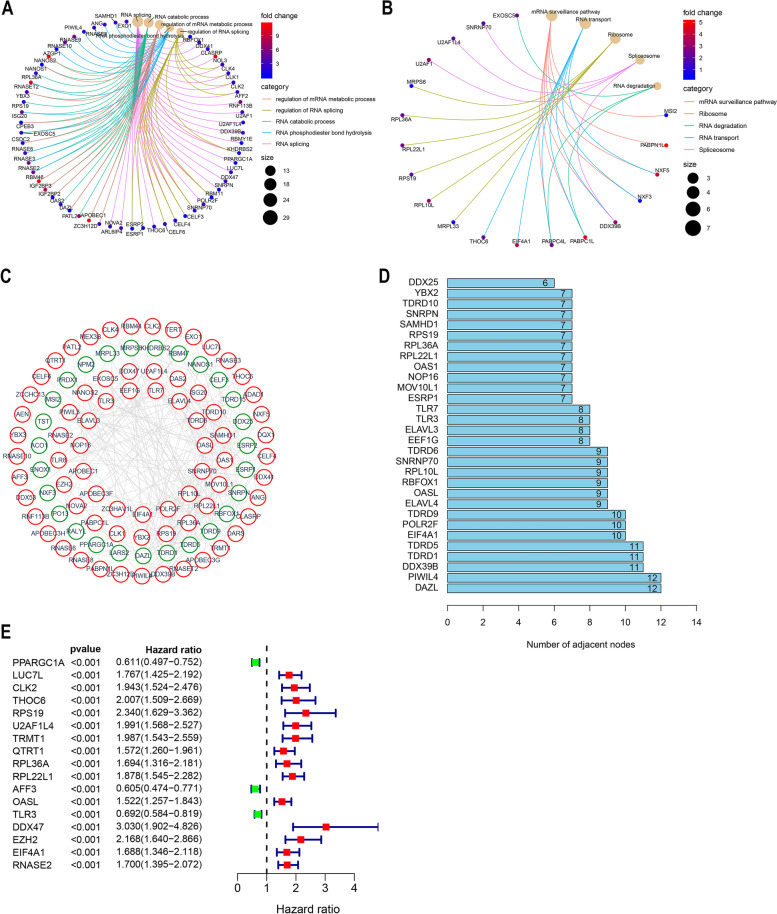
Bioenrichment analysis of differential expression RBPs. **A-B** Gene Ontology (GO) and Kyoto Encyclopedia of Genes and Genomes (KEGG) enrichment analysis, screening criteria as *p* < 0.05, q < 0.05 (Adjusted *p* value). DERBPs are enriched for relevant biological co-energies and associated molecular signalling pathways (BP:biological process CC: cell component MF: molecular function). **C** DERBPs protein–protein interaction network. The connections in the network represent interactions among genes, with green representing down-regulated genes and red up-regulated genes (Confidence = 0.7). **D** Number of node connections in a PPI network. The connections of the first 30 genes are displayed by the number of node connections. **E** RBPs associated with survival were screened by univariate Cox regression analysis (*p* < 0.0001)

We carried out GO and KEGG enrichment analysis for DERBPs. GO enrichment analysis includes three categories, including biological process (BP) analysis, cell component (CC) analysis and molecular function (MF) analysis. GO enrichment results showed that the enrichment of most genes was related to RNA metabolism and protein formation. Such as: “regulation of mRNA metabolic process," "regulation of RNA splicing," "RNA catabolic process," "RNA phosphodiester bond hydrolysis” and "RNA splicing". Figure 3A shows the GO enrichment results, with each function showing only the top 10 critical terms. KEGG enrichment analysis results showed that it was related to "Ribosome," "RNA transport," and "mRNA surveillance pathway." The analysis results are shown in Fig. 3B.

### Construction of protein–protein interaction networks

To explore the interaction between DERBPs, we used STRING, an online web page analysis tool, to build a PPI network (Confidence = 0.7). Which is visualized by using Cytoscape (Fig. 3C). Our PPI network contains 225 networks and 100 nodes. We only show the first 30 nodes based on the number of genes connected (Fig. 3D, Supplement Table 3).

### Prognostic related RBPs were screened

We performed univariate Cox regression analysis of RBPs in the network to screen genes associated with survival. The results showed that 17 genes (*p* < 0.0001) were correlated with the survival of ccRCC (Fig. 3E, Supplementary Fig. 1).

### Construction of the RBPs prognostic risk model

**Fig. 4:**
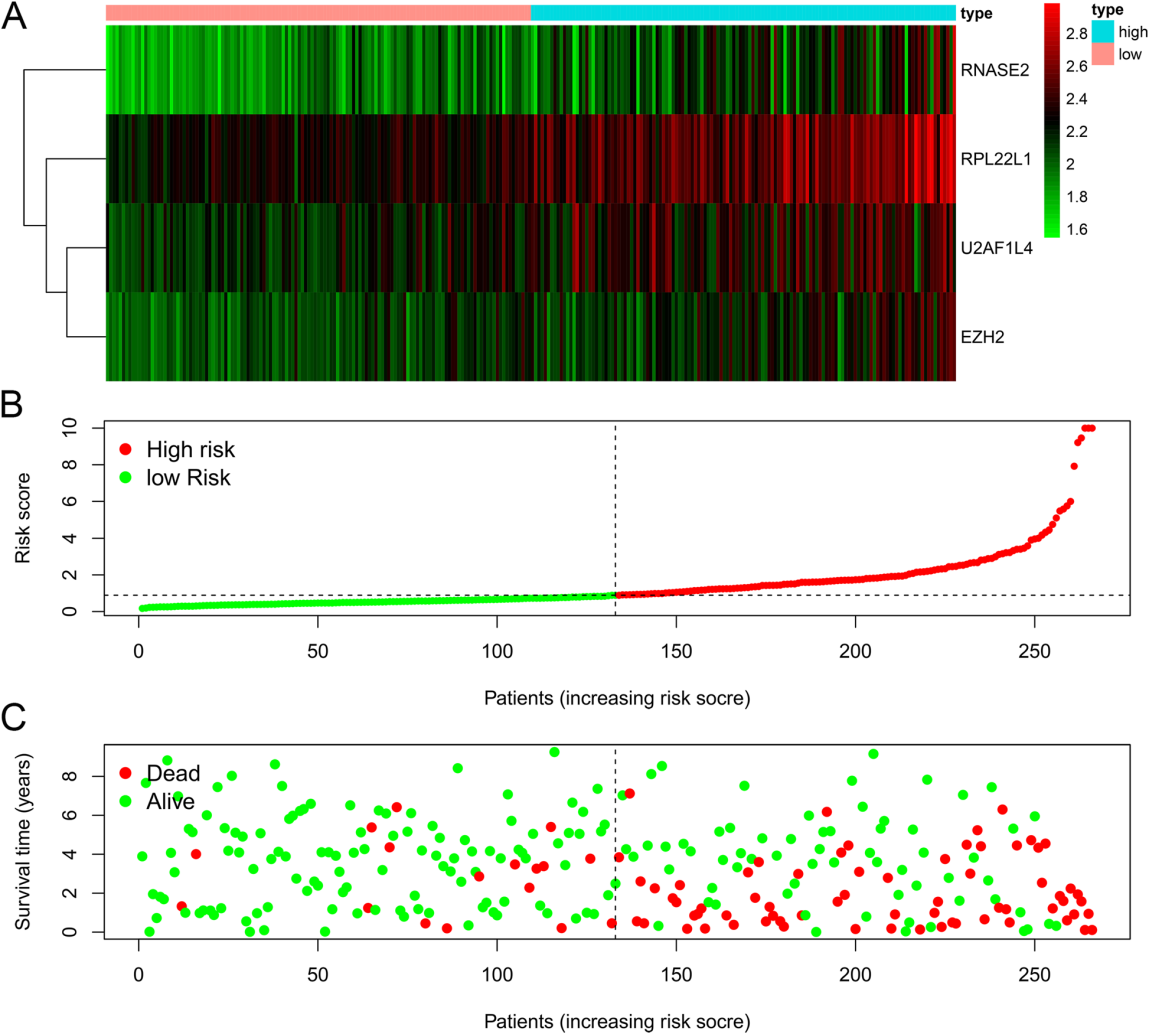
RBPs risk model. Multivariate Cox regression analyses were conducted on genes associated with survival and risk prediction models for RBPs were constructed. **A** The heat map of four genes (RNASE2, RPL22L1, U2AF1L4, and EZH2) expression (**B**) Distribution of risk score in RBPs model. **C** Survival chart of ccRCC patients

**Fig. 5:**
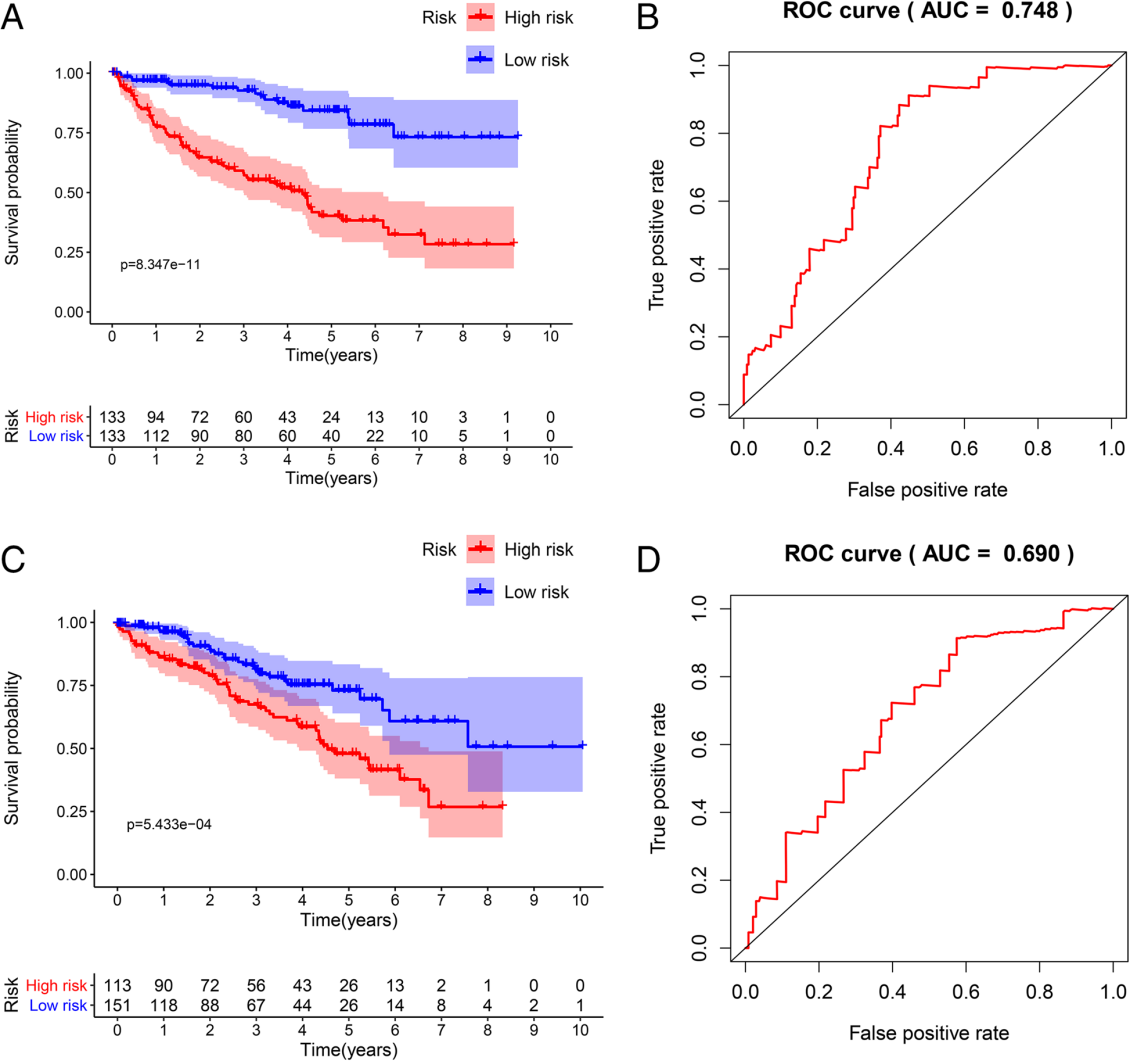
Validation of the model in the training and testing groups. Based on the median risk score, patients in each group were divided into high and low risk groups and Kaplan–Meier survival analysis was performed to see the significance of survival between the high and low risk groups. **A** The survival of high-risk and low-risk groups in the model. **B** The time-dependent ROC curve shows the area under curve (AUC) for ccRCC at 5 years (training group). **C**) Survival of high-risk and low-risk groups in the testing group. **D** The time-dependent ROC curve shows the area under curve (AUC) for ccRCC at 5 years (testing group)

In the training group, we used the built-in functions in R to perform a multivariate Cox regression analysis of 17 genes associated with survival, assessing the relative effect of each gene, comparing the regression *p*-values of individual genes, resulting in the identification of a four RBPs-related (U2AF1L4, RPL22L1, EZH2, RNASE2) prediction model (Fig. 4, Table 3). The patient's risk score was calculated according to the model, and the risk formula was as follows: Risk score = (EXP_U2AF1L4_ Χ 0.4984) + (EXP_RPL22L1_ X 0.5104) + (EXP_EZH2_ X 0.4014) + EXP_RNASE2_ X 0.6551). We divided patients into high-risk and low-risk groups based on the median risk score in the training data set. Kaplan–Meier survival analysis was performed to see the significance of survival between the high and low risk groups. We plotted survival curves to assess survival differences between the two groups, and we plotted the ROC curve to see the accuracy of our model predictions. The results showed that the five-year survival rate for the high-risk group was 40.2% (95% CI = 0.313 ~ 0.518), while the five-year survival rate for the low-risk group was 84.3% (95% CI = 0.767 ~ 0.926). The five-year survival rate for the low-risk group was much higher than that of the high-risk group (Fig. 5A). The ROC curves (AUC = 0.748) also showed that our model had stable predictive power (Fig. 5B).

**Table 3:** Multivariate Cox regression analysis of risk model prognosis-related RBPs

Gene	Coef	HR	*P*-value
U2AF1L4	0.498	1.646(1.111 ~ 2.439)	0.0123
RPL22L1	0.510	1.666(1.255 ~ 2.211)	< 0.0001
EZH2	0.401	1.494(0.971 ~ 2.298)	0.068
RNASE2	0.655	1.925(1.438 ~ 2.577)	< 0.0001

### Validation of the predictive power of risk model

To verify the predictive power of the model, we validated our model in the test group. Like the above risk formula, patients in the verification group were divided into high-risk and low-risk groups according to the median risk score. The survival and prognosis of the two groups were observed. The results in Fig. 5C showed that the high-risk group had a worse prognosis than the low-risk group. The five-year survival rate for the high-risk group was 47.9% (95% CI = 0.381 ~ 0.603), while the five-year survival rate for the low-risk group was 73% (95% CI = 0.639 ~ 0.834). The ROC analysis showed an AUC = 0.690, also demonstrating that our model also had good predictive power in the test group (Fig. 5D).

### Expression of model-related genes in tissues and cell lines

**Fig. 6:**
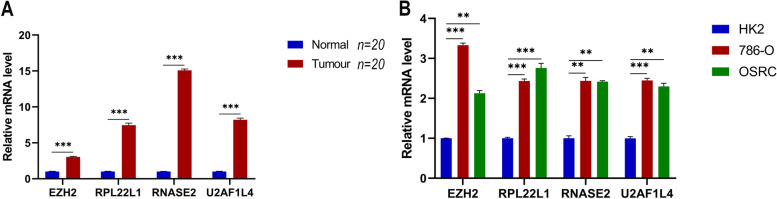
Expressions of hub genes were verified using RT-qPCR in 20 pairs of sample tissues and cell lines (HK2, 786-O, OSRC). This analysis was all compared using t-tests. The analysis results *p* < 0.05 considered the differences to be statistically significant. **, *p* < 0.01, ***, *p* < 0.001

This analysis was all compared using t-tests. The analysis results *p* < 0.05 considered the differences to be statistically significant. The RT-qPCR showed that the mRNA expressions of EZH2, RPL22L1, RNASE2, and U2AF1L4 were higher in renal tumor tissues than in normal renal tissues (Fig. 6A). In addition, this pattern was verified at the cellular level that the expressions of EZH2, RPL22L1, RNASE2, and U2AF1L4 were higher in renal tumor cells (786-O, OSRC) than in non-cancerous renal cells (HK2) (Fig. 6B). Collectively, these results demonstrated the accuracy of our model.

### Independent prognostic analysis and clinical correlation analysis

**Fig. 7:**
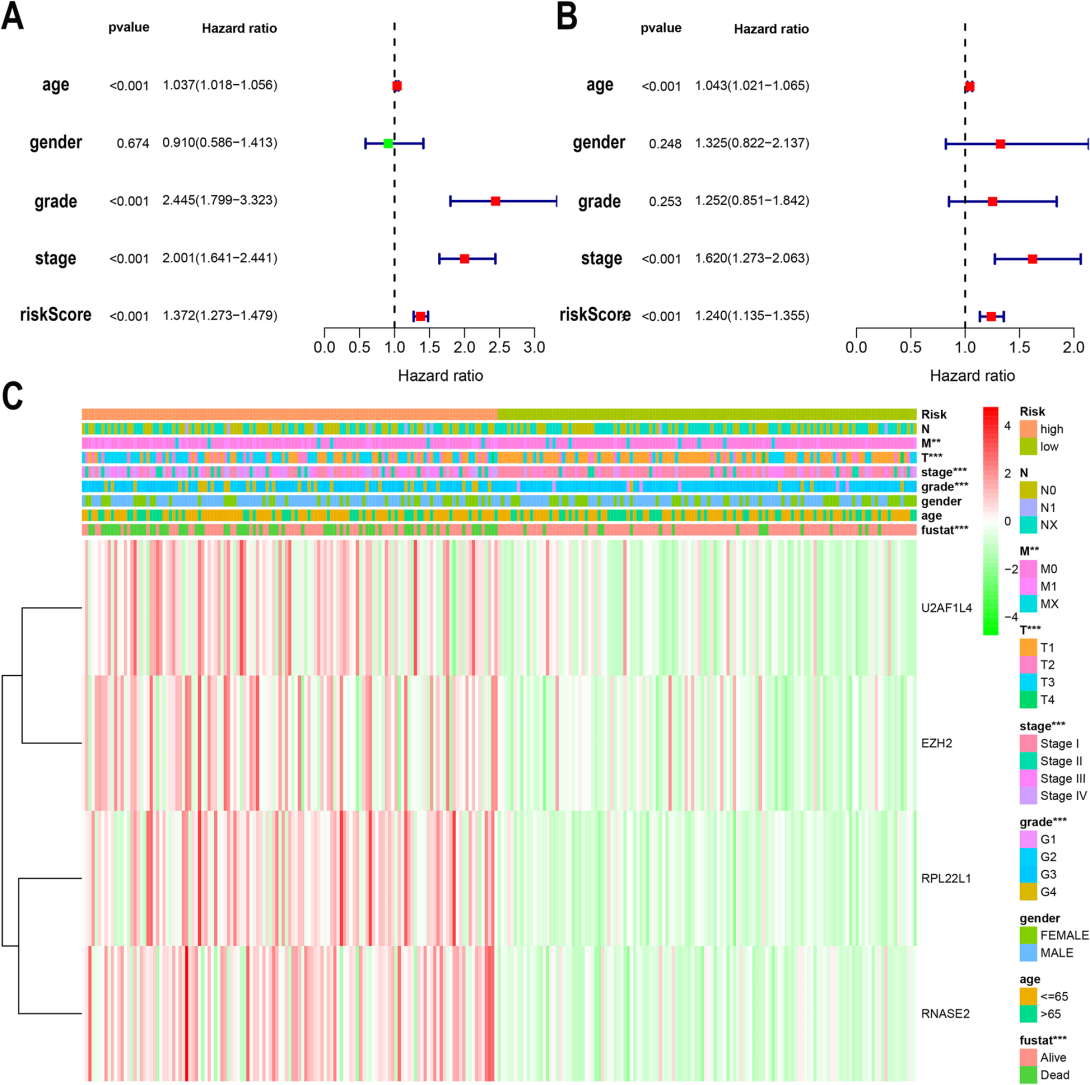
Prognostic analysis of the model and correlation between the model and clinicopathological indicators. **A** Univariate Cox regression analysis of the association between clinical parameters (including risk scores) and overall survival in patients with ccRCC. **B** Multivariate Cox regression analysis of the association between clinical parameters (including risk scores) and overall survival in patients with ccRCC. **C** Differences in clinicopathological indicators between the high and low risk groups. (clinicopathological indicators: AJCC stage, T stage, M stage, N stage, Grade, Gender, age)

**Fig. 8:**
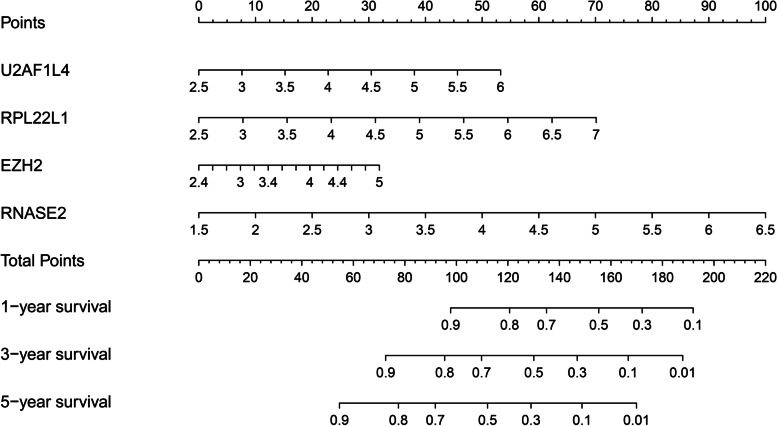
The clinical nomogram was developed to predict survival at 1, 3 and 5 years by incorporating risk model (U2AF1L4, RPL22L1, EZH2, RNASE2) metrics

To further evaluate whether our model can be used as a sole factor in assessing prognosis of ccRCC. Univariate and multivariate Cox regression analyses were performed for the model and clinical indicators. Univariate analysis (Fig. 7A) showed that patient age, tumor stage, tumor grade, and our model were associated with the prognosis of ccRCC patients (*p* < 0.001). Multivariate analysis (Fig. 7B) showed that patient age, tumor stage, and our model were associated with prognosis in ccRCC patients (*p* < 0.001). The results altogether demonstrated that our risk model could be used as an independent prognostic factor for ccRCC, and the accuracy of prediction is not affected by any other clinicopathological indicators. Chi-square test was used to analyze the correlation between the model and clinicopathological features. The results showed that the high-risk score was closely related to tumor stage (*p* < 0.001), pathological grade (*p* < 0.001), metastasis status (*p* < 0.01), but not related to gender or age (Fig. 7C). Also, the relationship between the expression levels of four genes in the model and the risk score was demonstrated. To better apply the model in the clinic, we developed the nomogram of the model. The 1-year, 3-year, and 5-year survival rates for ccRCC patients were predicted using the nomogram (Fig. 8).

## Discussion

Dysregulation of RBPs have been reported to be associated with tumor formation and progression [12, 20]. There have been several studies describing the prognostic value of RBPs in ccRCC [21–25]. For example, Hua et al. constructed a risk score model by using ten RBPs and validated the expression of hub genes in The Human Protein Altas database [20]; Xiang et al. also developed a risk model by using seven RBPs and potentially improve individualized diagnostic and therapeutic strategies of ccRCC [21]. However, the hub RBPs they identified and the risk models they built in above studies were not verified in real-world tumor tissues and cells. In order to make up for the shortcomings of previous studies, we built a new risk model of RBPs for predicting the survival of ccRCC and verified the expression of RBPs in clinical tissue samples and cancer cell lines. Moreover, the model we created consisted of only four RBPs, which makes our model more feasible when applying to clinical practice.

In this study, the ccRCC data were obtained from the TCGA database. We extracted the expression spectrum data based on 1542 RBPs and analyzed the differential expression of RBPs between the ccRCC group and the control group. We further performed GO and KEGG enrichment analysis for DERBPs and the results showed that DERBPs were enriched in the functions related to RNA metabolism, splicing, catabolic process and phosphodiester bond hydrolysis. Previous studies have shown that a variety of RBPs play important roles in the development and progression of kidney cancer. For example, Monocyte endoribonuclease acid endoribonuclease acid enzyme (MCPIP1) can affect the development of ccRCC by degrading the mRNA encoding pro-inflammatory cytokines [26]. In addition, there have been a large number of reports in the research of ccRCC that have proved the important correlation between RNA metabolism and the occurrence and development of tumors [27], and have provided the possibility for the precisely targeted therapy of ccRCC.

To construct a risk prediction model associated with RBPs, we performed univariate Cox analysis of genes in the PPI network to obtain RBPs associated with survival. We ended up with 17 genes (*p* < 0.0001) that were significantly associated with survival. We constructed a 4-RBPs risk model using multivariate Cox regression analysis of selected genes. Patients in the training group were divided into high-risk and low-risk groups according to the median risk score, and the results showed that the high-risk group had a worse prognosis than the low-risk group. The model results in the test group also showed that the high-risk group had a worse prognosis than the low-risk group, and the ROC curve results showed that our model had a stable predictive ability.

Our risk model consists of four RBPs genes, including U2AF1L4, RPL22L1, EZH2 and RNASE2. We found that all the genes in the model were risk factors for ccRCC. RNASE2 is an RBPs and immune-related gene that can be used as a marker of the immune risk model to predict patients' survival prognosis in ccRCC [28]. Studies have shown that EZH2 gene disorders or direct and indirect effects of other molecules can lead to the occurrence, development and metastasis of ccRCC [29, 30]. Besides, high expression of EZH2 correlates with poor prognosis in ccRCC. Our PCR results show that EZH2 gene expression levels are elevated in renal tumors compared to normal kidney tissue. It has been reported that RPL22L1 can be used as a marker in the ccRCC prediction model to predict the prognosis of patients [31]. Furthermore, RPL22L1 has been reported in other tumors. RPL22L1 can promote ovarian cancer metastasis by inhibiting vimentin and N-cadherin expression, thereby inducing epithelial-mesenchymal transition [32]. In addition, RPL22L1 could be used as a prognostic marker for prostate cancer and colorectal cancer [33, 34]. As a shear factor, U2AF1L4 plays an essential role in protein synthesis [35], however, U2AF1L4 has not been reported in ccRCC so far. More importantly, the differential expression of the four RBPs were verified in our clinical samples and cancer cell lines.

A comprehensive analysis of the model and clinicopathological features showed that the model was correlated with clinical stage, pathological grade and metastasis status. It is worth emphasizing that no correlation was found between the model and lymph node status, which was not in line with our expectations. After analysis, it turned out that the missing cases of lymph node status was more than 1/3 of the total cases, resulting in the deviation of the results. Cox regression analysis of our model and clinicopathological data proved that the model could be an independent prognostic factor for ccRCC and was not affected by clinical indicators. Finally, we constructed a nomogram to help clinicians predict 1-year, 3-year, and 5-year survival more accurately.

Our research still has some limitations. Firstly, the raw data are obtained from the TCGA database, and the patients in the database are mainly from America. The predictive ability of the model for patients from other countries needs to be further studied and confirmed. Secondly, although we have verified the model, subsequent clinical experiments are still needed to predict our model's predictive efficacy. Only in this way can our model be truly applied to the clinic and improve the prognosis prediction of ccRCC patients.

## Conclusions

In conclusion, we analyzed the role of RBPs in ccRCC, built a risk prediction model with RBPs, and verified the model's prediction efficiency. The model could be used to stratify patients with different prognosis and improve the clinical practice in the future.

## Supplementary Information

**Additional file 1: Supplement Table 1. **The 1542 RBPs genes in human cells.

**Additional file 2: Supplement Table 2. **Results of differential expression analysis of RBPs in the TCGA database ccRCC.

**Additional file 3: Supplement Table 3: **Number of nodes in the PPI network.

**Additional file 4: Supplementary Figure 1. **Survival analysis of 17 survival-related RBPs in ccRCC.

**Additional file 5. **RNA extraction from tissues.

## Data Availability

The data for constructing model was obtained from the TCGA database (https://portal.gdc.cancer.gov/) which is publicly available.

## Abbreviations

ccRCC: Clear cell renal carcinoma
RBPS: RNA binding proteins
TCGA: The Cancer Genome Atlas
DERBPS: Differentially expressed RBPs
RT-qPCR: Transcription-quantitative PCR
PPI: Protein–protein interactions
GO: Gene Ontology
KEGG: Kyoto Encyclopedia of Genes and Genomes
ROC: Receiver operating characteristic
BP: Biological process
CC: Cell component
MF: Molecular function
