# Correction: Construction and validation of a prognostic model of RNA binding proteins in clear cell renal carcinoma

**DOI:** 10.1186/s12882-022-02867-8

**Published:** 2022-07-15

**Authors:** Wenkai Han, Bohao Fan, Yongsheng Huang, Xiongbao Wang, Zhao Zhang, Gangli Gu, Zhao Liu

**Affiliations:** 1 Department of Clinical Medicine, Qingdao University, Qingdao, 266000 Shandong China; 2 Department of Urology, Qilu Hospital of Shandong University, Jinan, 250012 Shandong China

**Correction: BMC Nephrol 23, 172 (2022)**

**https://doi.org/10.1186/s12882-022-02801-y**

Following publication of the original article [1], the authors identified an error in the author name of Yongsheng Huang.

The incorrect author name is: Yongshen Huang

The correct author name is: Yongsheng Huang

The author group has been updated above and the original article [1] has been corrected.
