# Supplementary material for: Construction and validation of a prognostic model of RNA binding proteins in clear cell renal carcinoma
Source: BMC Nephrol. 2022 May 5;23:172. doi: 10.1186/s12882-022-02801-y (PMC9069774; doi:10.1186/s12882-022-02801-y)
Supplement: Supplementary file 1 — Additional file 1: Supplement Table 1. The 1542 RBPs genes in human cells. [file 12882_2022_2801_MOESM1_ESM.docx]

| **Supplement Table 1:** The 1542 RBPs genes in human cells. | |
| --- | --- |
| Gene Name |  |
| RPS11 |  |
| ERAL1 |  |
| DDX27 |  |
| DEK |  |
| PSMA6 |  |
| TRIM56 |  |
| TRIM71 |  |
| UPF2 |  |
| FARS2 |  |
| FDXACB1 |  |
| ALKBH8 |  |
| ZNF579 |  |
| POLR2B |  |
| WDR12 |  |
| RPF1 |  |
| MRPS34 |  |
| UBTF |  |
| CDC5L |  |
| DAZAP1 |  |
| MSI1 |  |
| MSI2 |  |
| TARDBP |  |
| TRIT1 |  |
| MRPL36 |  |
| KPNB1 |  |
| ZNFX1 |  |
| SNRNP27 |  |
| RBMS2 |  |
| RBMS1 |  |
| RBMS3 |  |
| LARS2 |  |
| KIAA0020 |  |
| RBFOX1 |  |
| RBFOX2 |  |
| RBFOX3 |  |
| CWF19L1 |  |
| CWF19L2 |  |
| ZC3H15 |  |
| LONP1 |  |
| DBR1 |  |
| RRP15 |  |
| L1TD1 |  |
| NMD3 |  |
| SRSF10 |  |
| SRSF12 |  |
| SRSF2 |  |
| SRSF8 |  |
| RSL1D1 |  |
| DAP3 |  |
| NARS2 |  |
| RBM25 |  |
| POLR2E |  |
| TOE1 |  |
| TSFM |  |
| TPR |  |
| STAU1 |  |
| STAU2 |  |
| TRMT1L |  |
| METTL3 |  |
| REXO2 |  |
| MRPL52 |  |
| WDR3 |  |
| CNOT11 |  |
| YTHDC1 |  |
| METTL2A |  |
| METTL2B |  |
| ZNF326 |  |
| AKAP8 |  |
| AKAP8L |  |
| RPS14 |  |
| ELAVL1 |  |
| ELAVL2 |  |
| ELAVL3 |  |
| ELAVL4 |  |
| PNN |  |
| DDX41 |  |
| TNPO2 |  |
| TNPO1 |  |
| PUSL1 |  |
| RBM43 |  |
| ZGPAT |  |
| SCAF11 |  |
| USP39 |  |
| RPL38 |  |
| NARS |  |
| RPS16 |  |
| CLASRP |  |
| RPS9 |  |
| TOP3B |  |
| NCBP2L |  |
| NCBP2 |  |
| SMG9 |  |
| FTSJ2 |  |
| DDX56 |  |
| RBM28 |  |
| UTP11L |  |
| MRPS18B |  |
| RPL14 |  |
| RPL37 |  |
| GEMIN5 |  |
| MKRN1 |  |
| MKRN2 |  |
| MKRN3 |  |
| NOA1 |  |
| PINX1 |  |
| HRSP12 |  |
| TDRD9 |  |
| DHX30 |  |
| DHX29 |  |
| DHX57 |  |
| YTHDC2 |  |
| DHX36 |  |
| DHX9 |  |
| EIF3G |  |
| NOL10 |  |
| UTP15 |  |
| GATC |  |
| TCOF1 |  |
| NONO |  |
| PSPC1 |  |
| SFPQ |  |
| NUDT21 |  |
| MRPL19 |  |
| URM1 |  |
| MRPL53 |  |
| RBM48 |  |
| REPIN1 |  |
| ZC3H12B |  |
| ZC3H12C |  |
| ZC3H12D |  |
| ZC3H12A |  |
| TRMT5 |  |
| TRMT61A |  |
| PABPN1L |  |
| PABPN1 |  |
| LSG1 |  |
| GNL1 |  |
| EEF1G |  |
| RBM33 |  |
| RPP25 |  |
| RPP25L |  |
| RPP38 |  |
| GARS |  |
| DRG2 |  |
| TRMU |  |
| GNL2 |  |
| CPSF2 |  |
| CD3EAP |  |
| HDLBP |  |
| RDM1 |  |
| BYSL |  |
| GEMIN8 |  |
| XPO5 |  |
| ZNF622 |  |
| DZIP1 |  |
| DZIP1L |  |
| GSPT1 |  |
| HBS1L |  |
| GSPT2 |  |
| EIF2D |  |
| METTL1 |  |
| RNASEH1 |  |
| ZNHIT6 |  |
| DARS2 |  |
| SNRPD2 |  |
| DICER1 |  |
| DYNLL1 |  |
| ZNF638 |  |
| SAMD4B |  |
| SAMD4A |  |
| RTF1 |  |
| CNOT4 |  |
| TARBP2 |  |
| PRKRA |  |
| GLE1 |  |
| EXOSC2 |  |
| SSU72 |  |
| GTPBP10 |  |
| RPL10A |  |
| HENMT1 |  |
| THOC2 |  |
| ENDOG |  |
| EXOG |  |
| ASH1L |  |
| TRMT10C |  |
| TRMT10A |  |
| TRMT10B |  |
| MRPL33 |  |
| SSB |  |
| LARP7 |  |
| MRPL24 |  |
| RNASEK |  |
| PELO |  |
| QARS |  |
| IPO4 |  |
| CAPRIN1 |  |
| CAPRIN2 |  |
| PNLDC1 |  |
| PARN |  |
| TIAL1 |  |
| TIA1 |  |
| CDC40 |  |
| ZNF598 |  |
| RCL1 |  |
| RTCA |  |
| MRPL38 |  |
| ADAT2 |  |
| BOP1 |  |
| U2SURP |  |
| DUS3L |  |
| CSTF3 |  |
| SRSF11 |  |
| SREK1 |  |
| POLR1E |  |
| EXOSC7 |  |
| EXOSC8 |  |
| EXOSC9 |  |
| MRPL12 |  |
| RPS28 |  |
| TEFM |  |
| RPL12 |  |
| ZC3H3 |  |
| EIF6 |  |
| NELFE |  |
| EIF2B3 |  |
| APOBEC2 |  |
| APOBEC3H |  |
| APOBEC3F |  |
| APOBEC3G |  |
| APOBEC1 |  |
| ZC3H7A |  |
| ZC3H7B |  |
| NOP9 |  |
| PQBP1 |  |
| PATL2 |  |
| PATL1 |  |
| EDC3 |  |
| WBP4 |  |
| GLTSCR2 |  |
| RPS18 |  |
| ASCC1 |  |
| EIF5A |  |
| EIF5A2 |  |
| EIF5AL1 |  |
| MRPL47 |  |
| BOLL |  |
| DAZL |  |
| DAZ1 |  |
| DAZ2 |  |
| DAZ3 |  |
| DAZ4 |  |
| INTS1 |  |
| RPLP1 |  |
| MRPS11 |  |
| WDR43 |  |
| TRMT112 |  |
| PPP1R8 |  |
| FBXO17 |  |
| MTHFSD |  |
| SON |  |
| SPATS2L |  |
| SPATS2 |  |
| MAEL |  |
| RRP1B |  |
| RRP1 |  |
| ZC3HAV1 |  |
| PARP12 |  |
| TIPARP |  |
| MRPS17 |  |
| U2AF2 |  |
| UHMK1 |  |
| NUP153 |  |
| CSDE1 |  |
| POLR2L |  |
| NKRF |  |
| MRPL48 |  |
| XRCC6 |  |
| SUPT5H |  |
| TSR3 |  |
| XRN2 |  |
| RSRC1 |  |
| TDRD5 |  |
| POLR2J |  |
| POLR2J2 |  |
| POLR2J3 |  |
| SAFB2 |  |
| SAFB |  |
| SLTM |  |
| BRIX1 |  |
| POLR2I |  |
| PWP2 |  |
| ELAC2 |  |
| ZCCHC14 |  |
| ZCCHC2 |  |
| LARS |  |
| MRPL28 |  |
| CNOT1 |  |
| DDX18 |  |
| TSEN15 |  |
| USP10 |  |
| SMNDC1 |  |
| THUMPD2 |  |
| THUMPD3 |  |
| SND1 |  |
| LSM4 |  |
| EIF3M |  |
| MRPL34 |  |
| SRP72 |  |
| SUGP1 |  |
| SUGP2 |  |
| RPS3 |  |
| RPS27A |  |
| CCAR2 |  |
| CCAR1 |  |
| DDX49 |  |
| MRPL4 |  |
| SFSWAP |  |
| MRPS12 |  |
| EIF1 |  |
| EIF1B |  |
| CLP1 |  |
| ZMAT5 |  |
| SF3B3 |  |
| PLD6 |  |
| QTRTD1 |  |
| QTRT1 |  |
| PIH1D1 |  |
| SWT1 |  |
| EXOSC4 |  |
| EXOSC6 |  |
| PTRH1 |  |
| INTS8 |  |
| EXOSC10 |  |
| CASC3 |  |
| GTF3A |  |
| MRPL54 |  |
| GTPBP3 |  |
| UBA1 |  |
| LSM12 |  |
| HINT3 |  |
| CCDC59 |  |
| POLDIP3 |  |
| RPL32 |  |
| AZGP1 |  |
| EMG1 |  |
| C17orf85 |  |
| N4BP1 |  |
| NYNRIN |  |
| KHNYN |  |
| NOP2 |  |
| NSUN4 |  |
| NSUN3 |  |
| NSUN6 |  |
| PAN2 |  |
| ZCCHC9 |  |
| RRP9 |  |
| TNRC6A |  |
| TNRC6B |  |
| TNRC6C |  |
| RP9 |  |
| MRPL15 |  |
| NGDN |  |
| LSM10 |  |
| PUS1 |  |
| MAK16 |  |
| RPS13 |  |
| SNRNP200 |  |
| PRDX1 |  |
| TSN |  |
| NUFIP2 |  |
| RBM19 |  |
| TDRKH |  |
| NOP58 |  |
| RBM6 |  |
| RBM10 |  |
| RBM5 |  |
| RBM44 |  |
| OASL |  |
| OAS1 |  |
| OAS2 |  |
| OAS3 |  |
| SF3A3 |  |
| NOC3L |  |
| MYEF2 |  |
| HNRNPM |  |
| C11orf68 |  |
| MRPL10 |  |
| RNASE10 |  |
| CALR |  |
| CANX |  |
| CALR3 |  |
| RPP14 |  |
| RPS8 |  |
| PDCD7 |  |
| RPS19BP1 |  |
| TRMT6 |  |
| RPL4 |  |
| PRPF3 |  |
| RARS |  |
| RARS2 |  |
| APOBEC4 |  |
| ZCCHC24 |  |
| RPS21 |  |
| DDX24 |  |
| RTCB |  |
| MBNL2 |  |
| MBNL3 |  |
| MBNL1 |  |
| ALYREF |  |
| LSMD1 |  |
| PUS7 |  |
| PUS7L |  |
| MRPS24 |  |
| RBBP6 |  |
| RPL19 |  |
| NOL3 |  |
| RPS27 |  |
| RPS27L |  |
| PTRF |  |
| MRPS18A |  |
| SLBP |  |
| EARS2 |  |
| SMAD1 |  |
| SMAD2 |  |
| SMAD5 |  |
| SMAD9 |  |
| SMAD3 |  |
| SMAD4 |  |
| SMAD7 |  |
| SMAD6 |  |
| PRPF39 |  |
| SRP68 |  |
| ADARB2 |  |
| ADAD1 |  |
| ADAD2 |  |
| ADAT1 |  |
| ADARB1 |  |
| ADAR |  |
| CLK3 |  |
| CLK4 |  |
| PRPF4B |  |
| CLK1 |  |
| CLK2 |  |
| LSM3 |  |
| FAM46A |  |
| CRYZ |  |
| KIN |  |
| RPS2 |  |
| DUS4L |  |
| EIF4A1 |  |
| EIF4A2 |  |
| EIF4A3 |  |
| IGF2BP1 |  |
| IGF2BP2 |  |
| IGF2BP3 |  |
| UBAP2 |  |
| UBAP2L |  |
| SNRPE |  |
| EIF2B5 |  |
| CRNKL1 |  |
| POLRMT |  |
| METTL10 |  |
| MRPL41 |  |
| MRPS7 |  |
| NSUN5 |  |
| NSUN7 |  |
| MTG1 |  |
| MRPS28 |  |
| TDRD10 |  |
| RPUSD1 |  |
| NHP2 |  |
| XAB2 |  |
| FAU |  |
| RRP36 |  |
| GRWD1 |  |
| ENDOV |  |
| VARSL |  |
| MRRF |  |
| MTFMT |  |
| C12orf65 |  |
| MRTO4 |  |
| RBM26 |  |
| RBM27 |  |
| DIS3 |  |
| DIS3L |  |
| DIS3L2 |  |
| C1QBP |  |
| SUB1 |  |
| MRPS33 |  |
| PRPF6 |  |
| SMG8 |  |
| PSTK |  |
| GAPDH |  |
| TNPO3 |  |
| IPO13 |  |
| MOV10 |  |
| MOV10L1 |  |
| PARP1 |  |
| MRPS35 |  |
| GPKOW |  |
| SETD1A |  |
| SETD1B |  |
| SF3A2 |  |
| AFF1 |  |
| AFF3 |  |
| AFF4 |  |
| AFF2 |  |
| PARK7 |  |
| TRNAU1AP |  |
| SRBD1 |  |
| EIF3D |  |
| NOP10 |  |
| WARS |  |
| MATR3 |  |
| SNRPD1 |  |
| SNRPC |  |
| FXR1 |  |
| FXR2 |  |
| FMR1 |  |
| MEPCE |  |
| KAT8 |  |
| CIRBP |  |
| RBM3 |  |
| CNOT7 |  |
| CNOT8 |  |
| A1CF |  |
| RBM46 |  |
| RBM47 |  |
| DND1 |  |
| HNRNPR |  |
| SYNCRIP |  |
| ABCE1 |  |
| GPATCH4 |  |
| PAIP1 |  |
| RBM20 |  |
| GNL3 |  |
| GNL3L |  |
| FAM120A |  |
| FAM120B |  |
| FAM120C |  |
| KHDC1 |  |
| KHDC1L |  |
| RPL10L |  |
| RPL10 |  |
| EIF2S1 |  |
| MRPL20 |  |
| EPRS |  |
| DHX58 |  |
| DDX58 |  |
| IFIH1 |  |
| CDK5RAP1 |  |
| HABP4 |  |
| SERBP1 |  |
| METTL5 |  |
| ZCCHC7 |  |
| WDR36 |  |
| CCDC86 |  |
| C2orf15 |  |
| MRPL30 |  |
| RPS26 |  |
| TEX13A |  |
| BARD1 |  |
| XPO4 |  |
| RUVBL1 |  |
| IMP4 |  |
| DDX52 |  |
| THOC1 |  |
| TRPT1 |  |
| NHP2L1 |  |
| EIF2B2 |  |
| RNF113B |  |
| RNF113A |  |
| INTS10 |  |
| LENG9 |  |
| RPS29 |  |
| NOP16 |  |
| U2AF1 |  |
| U2AF1L4 |  |
| RANBP6 |  |
| IPO5 |  |
| THOC7 |  |
| DNMT3B |  |
| GFM1 |  |
| RNASE9 |  |
| PLRG1 |  |
| REXO1 |  |
| ZC3HAV1L |  |
| LSM14A |  |
| LSM14B |  |
| LUZP4 |  |
| TDRD6 |  |
| TDRD15 |  |
| MRPS22 |  |
| EEF1B2 |  |
| EEF1D |  |
| SIDT1 |  |
| SIDT2 |  |
| TRMT44 |  |
| GPATCH8 |  |
| WDR4 |  |
| SLIRP |  |
| ATXN1 |  |
| ATXN1L |  |
| YTHDF1 |  |
| YTHDF2 |  |
| YTHDF3 |  |
| LRPPRC |  |
| TFIP11 |  |
| ILF2 |  |
| STRBP |  |
| ILF3 |  |
| TCERG1 |  |
| NXF5 |  |
| NXF2B |  |
| NXF2 |  |
| NXF1 |  |
| NXF3 |  |
| ZRSR1 |  |
| ZRSR2 |  |
| MRPL35 |  |
| RRS1 |  |
| PRKDC |  |
| NOC2L |  |
| MTIF3 |  |
| NOL11 |  |
| EXOSC3 |  |
| PNPT1 |  |
| TSNAX |  |
| RIOK1 |  |
| RIOK3 |  |
| PIWIL1 |  |
| PIWIL2 |  |
| PIWIL3 |  |
| PIWIL4 |  |
| PEG10 |  |
| ZCCHC5 |  |
| POP7 |  |
| FASTKD3 |  |
| TBRG4 |  |
| FASTK |  |
| TRMT13 |  |
| IREB2 |  |
| ACO1 |  |
| ZCCHC3 |  |
| SNUPN |  |
| PTRHD1 |  |
| HARS |  |
| HARS2 |  |
| HNRNPK |  |
| PCBP1 |  |
| PCBP2 |  |
| PCBP3 |  |
| PCBP4 |  |
| AQR |  |
| ICT1 |  |
| UNK |  |
| UNKL |  |
| CEBPZ |  |
| MRPS9 |  |
| THG1L |  |
| ZCCHC13 |  |
| CNBP |  |
| SRRT |  |
| CD2BP2 |  |
| SNRPG |  |
| APTX |  |
| RPL17 |  |
| BMS1 |  |
| SARS |  |
| SARS2 |  |
| PAIP2 |  |
| PAIP2B |  |
| GEMIN4 |  |
| PNRC2 |  |
| FASTKD2 |  |
| GEMIN6 |  |
| DDX20 |  |
| DDX39A |  |
| DDX39B |  |
| DHX37 |  |
| ZNF346 |  |
| MRPL50 |  |
| RNASE1 |  |
| RNASE2 |  |
| RNASE3 |  |
| RNASE4 |  |
| RNASE6 |  |
| RNASE7 |  |
| RNASE8 |  |
| ANG |  |
| DYNC1H1 |  |
| FTSJ3 |  |
| SF3B2 |  |
| CTU2 |  |
| XPOT |  |
| RBMXL1 |  |
| RBMXL2 |  |
| RBMXL3 |  |
| RBMY1A1 |  |
| RBMY1B |  |
| RBMY1D |  |
| RBMY1E |  |
| RBMY1F |  |
| RBMY1J |  |
| RBMX |  |
| PPP1R10 |  |
| PRR3 |  |
| DKC1 |  |
| MRPL17 |  |
| TRA2A |  |
| TRA2B |  |
| RPL26L1 |  |
| RPL26 |  |
| AUH |  |
| EIF3C |  |
| EIF3CL |  |
| SCAF8 |  |
| SCAF4 |  |
| EEF2K |  |
| RPL6 |  |
| MRPL11 |  |
| G3BP1 |  |
| G3BP2 |  |
| MRPS26 |  |
| SF3B5 |  |
| DHX34 |  |
| UTP23 |  |
| SNRNP48 |  |
| PRPF38B |  |
| HEXIM1 |  |
| HEXIM2 |  |
| SRRM2 |  |
| SRRM3 |  |
| PIH1D3 |  |
| DHX40 |  |
| DHX38 |  |
| TRMT61B |  |
| CSDC2 |  |
| CARHSP1 |  |
| POP4 |  |
| LARP6 |  |
| RNPC3 |  |
| RPL30 |  |
| RUVBL2 |  |
| DRG1 |  |
| TRIM25 |  |
| RQCD1 |  |
| MRPS18C |  |
| MRPL45 |  |
| RPLP2 |  |
| SRP19 |  |
| RPS24 |  |
| AKAP17A |  |
| NXT2 |  |
| NXT1 |  |
| MRPL9 |  |
| RPL27 |  |
| RPL24 |  |
| CNOT10 |  |
| ABT1 |  |
| DDX25 |  |
| DDX19A |  |
| DDX19B |  |
| MRPS30 |  |
| SMG1 |  |
| EXOSC5 |  |
| AIMP2 |  |
| BZW2 |  |
| BZW1 |  |
| PARS2 |  |
| INTS12 |  |
| EIF3H |  |
| SKIV2L |  |
| RC3H2 |  |
| RC3H1 |  |
| ZFP36 |  |
| ZFP36L1 |  |
| ZFP36L2 |  |
| ANKHD1 |  |
| ANKRD17 |  |
| HELZ2 |  |
| UPF1 |  |
| DROSHA |  |
| MRPL37 |  |
| PUS10 |  |
| NUDT16 |  |
| NUDT16L1 |  |
| TRMT11 |  |
| PET112 |  |
| NUFIP1 |  |
| PURA |  |
| PURB |  |
| PURG |  |
| ZRANB2 |  |
| THOC5 |  |
| TSEN54 |  |
| SRP9 |  |
| PIN4 |  |
| ZC3H14 |  |
| CNOT2 |  |
| TARBP1 |  |
| AGO1 |  |
| AGO2 |  |
| AGO3 |  |
| AGO4 |  |
| LARP4B |  |
| LARP4 |  |
| SNW1 |  |
| EZH2 |  |
| EIF3B |  |
| RPP30 |  |
| ZMAT3 |  |
| PTGES3 |  |
| KARS |  |
| RAN |  |
| SNRPA |  |
| SNRPB2 |  |
| HEATR1 |  |
| PTGES3L-AARSD1 |  |
| AARSD1 |  |
| CPSF3 |  |
| CPSF3L |  |
| CWC25 |  |
| RPS5 |  |
| POLR2D |  |
| C9orf114 |  |
| MRPL44 |  |
| ETF1 |  |
| GAR1 |  |
| MVP |  |
| RPF2 |  |
| ZC3H18 |  |
| INTS5 |  |
| INTS3 |  |
| LSM6 |  |
| SNRPF |  |
| TUT1 |  |
| MTPAP |  |
| EIF2AK1 |  |
| EIF2AK2 |  |
| EIF2AK3 |  |
| EIF2AK4 |  |
| MRPL22 |  |
| PES1 |  |
| LSM7 |  |
| PAPD4 |  |
| ZCCHC11 |  |
| ZCCHC6 |  |
| KHDRBS1 |  |
| KHDRBS2 |  |
| KHDRBS3 |  |
| QKI |  |
| SF1 |  |
| LRRC47 |  |
| ISY1 |  |
| SRRM1 |  |
| IPO9 |  |
| MTRF1 |  |
| MTRF1L |  |
| WIBG |  |
| PPAN |  |
| FUBP3 |  |
| FUBP1 |  |
| KHSRP |  |
| CHTOP |  |
| PTCD2 |  |
| POLR2A |  |
| MRPL43 |  |
| DDX55 |  |
| RPS15 |  |
| CPSF4 |  |
| CPSF4L |  |
| CSTF2 |  |
| CSTF2T |  |
| RAVER2 |  |
| RAVER1 |  |
| EIF1AX |  |
| EIF1AY |  |
| DALRD3 |  |
| NSUN2 |  |
| URB2 |  |
| PHAX |  |
| PPARGC1A |  |
| PPARGC1B |  |
| PPRC1 |  |
| PHF5A |  |
| MCTS1 |  |
| RRP7A |  |
| DGCR8 |  |
| ZC3H13 |  |
| UPF3A |  |
| UPF3B |  |
| RBM24 |  |
| RBM38 |  |
| THOC3 |  |
| IGHMBP2 |  |
| PAPD5 |  |
| PAPD7 |  |
| GFM2 |  |
| PHRF1 |  |
| VARS |  |
| VARS2 |  |
| MRPL1 |  |
| INTS7 |  |
| ATXN2L |  |
| ATXN2 |  |
| PPIL4 |  |
| PPIL3 |  |
| PPWD1 |  |
| CWC27 |  |
| PPIH |  |
| NUTF2 |  |
| MRPL18 |  |
| LSM11 |  |
| AARS2 |  |
| AARS |  |
| MIF4GD |  |
| CTIF |  |
| C9orf129 |  |
| LUC7L2 |  |
| LUC7L3 |  |
| LUC7L |  |
| DUS2 |  |
| RNF17 |  |
| TDRD1 |  |
| MRPS10 |  |
| FAM103A1 |  |
| ENOX1 |  |
| ENOX2 |  |
| RBPMS |  |
| RBPMS2 |  |
| NIP7 |  |
| RPL3 |  |
| RPL3L |  |
| RNASE11 |  |
| RNASE12 |  |
| RPLP0 |  |
| RBM34 |  |
| CMTR2 |  |
| ENDOU |  |
| PUM1 |  |
| PUM2 |  |
| TYW5 |  |
| TYW3 |  |
| TRMT1 |  |
| RAE1 |  |
| SLU7 |  |
| EIF2B4 |  |
| EIF5B |  |
| GPATCH1 |  |
| RPL36 |  |
| EEF1A1 |  |
| EEF1A2 |  |
| RRP8 |  |
| UBA52 |  |
| R3HDM1 |  |
| R3HDM2 |  |
| PA2G4 |  |
| AAR2 |  |
| ERI1 |  |
| ERI2 |  |
| ERI3 |  |
| NIFK |  |
| METTL14 |  |
| MRPL32 |  |
| CPSF6 |  |
| CPSF7 |  |
| RPL7A |  |
| WDR46 |  |
| SRP14 |  |
| TFAM |  |
| CMSS1 |  |
| XPO6 |  |
| EXOSC1 |  |
| ZNF106 |  |
| UTP14A |  |
| UTP14C |  |
| TRUB1 |  |
| TRUB2 |  |
| PRIM1 |  |
| PTCD3 |  |
| HNRNPA0 |  |
| HNRNPA1 |  |
| HNRNPA1L2 |  |
| HNRNPA2B1 |  |
| HNRNPA3 |  |
| HNRNPAB |  |
| HNRNPD |  |
| HNRNPDL |  |
| RANBP2 |  |
| RPL7 |  |
| RPL7L1 |  |
| RNASE13 |  |
| RPS6 |  |
| MRPL3 |  |
| DDX28 |  |
| DDX47 |  |
| RNASEH2C |  |
| TFB2M |  |
| TEP1 |  |
| NOC4L |  |
| CPSF1 |  |
| DDX1 |  |
| DDX3X |  |
| DDX3Y |  |
| DDX4 |  |
| NOP56 |  |
| RPS20 |  |
| MPHOSPH10 |  |
| MRM1 |  |
| DXO |  |
| DENR |  |
| RPL21 |  |
| RPS10 |  |
| NAT10 |  |
| UTP18 |  |
| NOL12 |  |
| DDX31 |  |
| ABCF1 |  |
| DNMT1 |  |
| SNRPA1 |  |
| API5 |  |
| SHQ1 |  |
| AKAP1 |  |
| FTO |  |
| PUF60 |  |
| SAMHD1 |  |
| RBM45 |  |
| FAM98B |  |
| FAM98A |  |
| FAM98C |  |
| PCF11 |  |
| EIF5 |  |
| ZC3HC1 |  |
| HNRNPC |  |
| HNRNPCL1 |  |
| RALY |  |
| RALYL |  |
| SEPSECS |  |
| NPM1 |  |
| NPM3 |  |
| NPM2 |  |
| NUPL2 |  |
| MRPS14 |  |
| SF3A1 |  |
| EIF3A |  |
| FASTKD5 |  |
| TDRD3 |  |
| SNRPB |  |
| SNRPN |  |
| YRDC |  |
| POLR2K |  |
| ERN2 |  |
| ERN1 |  |
| RPL5 |  |
| RPS17 |  |
| RPS17L |  |
| DARS |  |
| IPO7 |  |
| IPO8 |  |
| SMN1 |  |
| SMN2 |  |
| GTPBP4 |  |
| MRPL13 |  |
| MRPL46 |  |
| LSM5 |  |
| EIF4G3 |  |
| EIF4G1 |  |
| EIF4G2 |  |
| GTF2F1 |  |
| RPS15A |  |
| PIH1D2 |  |
| TTF2 |  |
| PUS3 |  |
| RBM7 |  |
| RBM11 |  |
| TSEN2 |  |
| RBM42 |  |
| UTP3 |  |
| HNRNPL |  |
| HNRNPLL |  |
| PTBP1 |  |
| PTBP2 |  |
| PTBP3 |  |
| SYMPK |  |
| POLR2F |  |
| TSR2 |  |
| KIAA0391 |  |
| POLR2G |  |
| WDR5 |  |
| SNRNP40 |  |
| WDR83 |  |
| ZFC3H1 |  |
| RPL15 |  |
| SNRNP35 |  |
| SNRNP70 |  |
| MRPL39 |  |
| TLR7 |  |
| TLR8 |  |
| TLR3 |  |
| NAA38 |  |
| PRPF31 |  |
| MRPL55 |  |
| TRMT2A |  |
| TRMT2B |  |
| RPS3A |  |
| BRCA1 |  |
| RIOK2 |  |
| DHX16 |  |
| DHX33 |  |
| DHX35 |  |
| DHX32 |  |
| DQX1 |  |
| DHX15 |  |
| DHX8 |  |
| MRPS5 |  |
| SAP18 |  |
| TUFM |  |
| EEFSEC |  |
| TDRD12 |  |
| SKIV2L2 |  |
| TRDMT1 |  |
| ZNF473 |  |
| FCF1 |  |
| URB1 |  |
| CARS |  |
| CARS2 |  |
| CELF1 |  |
| CELF2 |  |
| CELF3 |  |
| CELF4 |  |
| CELF5 |  |
| CELF6 |  |
| XPO1 |  |
| DDX17 |  |
| DDX46 |  |
| DDX5 |  |
| DDX42 |  |
| DDX43 |  |
| DDX53 |  |
| FIP1L1 |  |
| MRPL14 |  |
| TXNL4A |  |
| TXNL4B |  |
| RBM39 |  |
| RBM23 |  |
| CPEB1 |  |
| CPEB2 |  |
| CPEB3 |  |
| CPEB4 |  |
| INTS9 |  |
| CACTIN |  |
| MPHOSPH6 |  |
| RPL34 |  |
| OBFC1 |  |
| RPS12 |  |
| PRPF8 |  |
| SECISBP2L |  |
| SECISBP2 |  |
| RPUSD3 |  |
| RPUSD4 |  |
| DDX6 |  |
| RPL28 |  |
| ELAC1 |  |
| NOM1 |  |
| TROVE2 |  |
| EEF1E1 |  |
| TARSL2 |  |
| TARS |  |
| TARS2 |  |
| IARS |  |
| RPL18A |  |
| ESF1 |  |
| EEF2 |  |
| EFTUD2 |  |
| DDX60 |  |
| DDX60L |  |
| MRPL2 |  |
| SLC4A1AP |  |
| MAZ |  |
| EIF2S3L |  |
| EIF2S3 |  |
| AIMP1 |  |
| APEX1 |  |
| EIF3J |  |
| PABPC3 |  |
| PABPC1L |  |
| PABPC1L2A |  |
| PABPC1L2B |  |
| PABPC4L |  |
| PABPC5 |  |
| PABPC4 |  |
| PABPC1 |  |
| MEX3A |  |
| MEX3B |  |
| MEX3C |  |
| MEX3D |  |
| GEMIN7 |  |
| MRPS23 |  |
| PSIP1 |  |
| SRP54 |  |
| FARSB |  |
| SPEN |  |
| RBM15B |  |
| RBM15 |  |
| DNAAF2 |  |
| SF3B1 |  |
| RNASEH2B |  |
| FARSA |  |
| NOB1 |  |
| EIF2A |  |
| RNASEL |  |
| TRNT1 |  |
| EIF4ENIF1 |  |
| MECP2 |  |
| RBM22 |  |
| CMTR1 |  |
| RPL39 |  |
| RPL39L |  |
| RPSA |  |
| PARP4 |  |
| CSTF1 |  |
| NCBP1 |  |
| SNRNP25 |  |
| DNAJC21 |  |
| CWC22 |  |
| NFX1 |  |
| MRPS31 |  |
| SMG5 |  |
| SART1 |  |
| GEMIN2 |  |
| NR0B1 |  |
| DCP2 |  |
| TAF15 |  |
| EWSR1 |  |
| FUS |  |
| EDC4 |  |
| FBLL1 |  |
| FBL |  |
| MRPS27 |  |
| ALKBH1 |  |
| ZCCHC17 |  |
| MRPL49 |  |
| RPL18 |  |
| RPS4X |  |
| RPS4Y1 |  |
| RPS4Y2 |  |
| NOP14 |  |
| KRR1 |  |
| NCL |  |
| PRPF18 |  |
| C1D |  |
| ADAT3 |  |
| UTP20 |  |
| SNIP1 |  |
| GUF1 |  |
| GCFC2 |  |
| UTP6 |  |
| SRSF1 |  |
| SRSF3 |  |
| SRSF4 |  |
| SRSF5 |  |
| SRSF6 |  |
| SRSF7 |  |
| SRSF9 |  |
| USB1 |  |
| NOL6 |  |
| PPIE |  |
| RRBP1 |  |
| SUZ12 |  |
| TERT |  |
| MSL3 |  |
| BICC1 |  |
| MRPS16 |  |
| RBMX2 |  |
| MRPL40 |  |
| ISG20 |  |
| ISG20L2 |  |
| AEN |  |
| REXO4 |  |
| TGS1 |  |
| ZC3H4 |  |
| ZC3H6 |  |
| ZC3H8 |  |
| RPL27A |  |
| RPS19 |  |
| SYF2 |  |
| TFB1M |  |
| DIMT1 |  |
| TSEN34 |  |
| DCAF13 |  |
| RPL9 |  |
| ARHGEF28 |  |
| AC004381.6 |  |
| EIF4H |  |
| EIF4B |  |
| DDX54 |  |
| MRPS36 |  |
| PRPF40A |  |
| PRPF40B |  |
| EIF3L |  |
| NOL8 |  |
| MTO1 |  |
| PSMA1 |  |
| HTATSF1 |  |
| FTSJ1 |  |
| LAS1L |  |
| POP1 |  |
| DZIP3 |  |
| DGCR14 |  |
| CDK9 |  |
| PAN3 |  |
| RPL29 |  |
| DCPS |  |
| MRP63 |  |
| MRPL27 |  |
| POLR2H |  |
| ZNF239 |  |
| XRN1 |  |
| ZFR |  |
| ZFR2 |  |
| AGFG1 |  |
| NSA2 |  |
| DDX10 |  |
| TBL3 |  |
| INTS2 |  |
| YBX1 |  |
| YBX2 |  |
| YBX3 |  |
| SURF6 |  |
| THOC6 |  |
| EIF3I |  |
| STRAP |  |
| RPL13 |  |
| RRP12 |  |
| MRPS2 |  |
| ZCCHC8 |  |
| EFTUD1 |  |
| RPL35 |  |
| NSRP1 |  |
| RPUSD2 |  |
| BCDIN3D |  |
| THUMPD1 |  |
| TRIM21 |  |
| RANBP17 |  |
| XPO7 |  |
| TRMT12 |  |
| ACIN1 |  |
| ZNF385A |  |
| RPL23 |  |
| EED |  |
| DNTTIP2 |  |
| SBDS |  |
| DDX51 |  |
| RBM18 |  |
| IPO11 |  |
| RPL22 |  |
| RPL22L1 |  |
| LCMT2 |  |
| ZC3H11A |  |
| FYTTD1 |  |
| RNPS1 |  |
| RPS23 |  |
| RNASEH2A |  |
| FRG1B |  |
| FRG1 |  |
| NOL7 |  |
| PRPF38A |  |
| RBM41 |  |
| RBM12 |  |
| RBM12B |  |
| ESRP1 |  |
| ESRP2 |  |
| HNRNPF |  |
| HNRNPH1 |  |
| HNRNPH2 |  |
| HNRNPH3 |  |
| GRSF1 |  |
| RNH1 |  |
| NOL9 |  |
| SRFBP1 |  |
| ARL6IP4 |  |
| RPP40 |  |
| ALKBH5 |  |
| SMG7 |  |
| PDE12 |  |
| CCRN4L |  |
| ANGEL1 |  |
| ANGEL2 |  |
| CNOT6 |  |
| CNOT6L |  |
| PDCD11 |  |
| RPL35A |  |
| MRPL21 |  |
| SMG6 |  |
| PRPF19 |  |
| PRPF4 |  |
| INTS4 |  |
| SRPK2 |  |
| SUPV3L1 |  |
| RBM17 |  |
| NAF1 |  |
| YARS |  |
| LRRFIP1 |  |
| LRRFIP2 |  |
| EIF4E2 |  |
| EIF4E1B |  |
| EIF4E3 |  |
| EIF4E |  |
| BUD13 |  |
| CTU1 |  |
| GTPBP1 |  |
| GTPBP2 |  |
| MTERFD2 |  |
| SRPR |  |
| DDX50 |  |
| DDX21 |  |
| ZCRB1 |  |
| RPL13A |  |
| SRA1 |  |
| PAPOLB |  |
| PAPOLG |  |
| PAPOLA |  |
| RPL41 |  |
| ZNF768 |  |
| SCAF1 |  |
| MRPS15 |  |
| RNASET2 |  |
| EIF2B1 |  |
| RPL36A |  |
| RPL36AL |  |
| DDX26B |  |
| INTS6 |  |
| RPS7 |  |
| RRNAD1 |  |
| MRPS21 |  |
| SETD7 |  |
| RBM8A |  |
| MAGOH |  |
| MAGOHB |  |
| RPL37A |  |
| NOLC1 |  |
| IARS2 |  |
| LSM1 |  |
| SUPT4H1 |  |
| EXO1 |  |
| DCP1B |  |
| DCP1A |  |
| DNAJC17 |  |
| RNGTT |  |
| DUSP11 |  |
| CHERP |  |
| WDR61 |  |
| NANOS1 |  |
| NANOS2 |  |
| NANOS3 |  |
| EBNA1BP2 |  |
| HELZ |  |
| RNMTL1 |  |
| PDCD4 |  |
| PTRH2 |  |
| SART3 |  |
| ZC3H10 |  |
| LSM2 |  |
| MRPS25 |  |
| RPP21 |  |
| ZMAT2 |  |
| IFIT1 |  |
| IFIT2 |  |
| IFIT5 |  |
| IFIT1B |  |
| IFIT3 |  |
| NOVA1 |  |
| NOVA2 |  |
| SNRPD3 |  |
| YARS2 |  |
| PTCD1 |  |
| TSR1 |  |
| MRPS6 |  |
| TAF9 |  |
| RPL11 |  |
| DDX23 |  |
| DDX59 |  |
| CCNT2 |  |
| CCNT1 |  |
| RPL8 |  |
| SARNP |  |
| IMP3 |  |
| SRRM4 |  |
| CNP |  |
| LIN28A |  |
| LIN28B |  |
| EIF3K |  |
| SF3B14 |  |
| EIF2S2 |  |
| RNMT |  |
| MRPL42 |  |
| THRAP3 |  |
| BCLAF1 |  |
| CXorf23 |  |
| PNO1 |  |
| RBM4 |  |
| RBM4B |  |
| RBM14 |  |
| R3HCC1 |  |
| R3HCC1L |  |
| KIAA0430 |  |
| POP5 |  |
| MRPL51 |  |
| MARS |  |
| TYW1 |  |
| MRPL16 |  |
| DUS1L |  |
| QRSL1 |  |
| WARS2 |  |
| WRAP53 |  |
| RSL24D1 |  |
| SF3B4 |  |
| TDRD7 |  |
| LARP1 |  |
| LARP1B |  |
| RPL23A |  |
| BAZ2A |  |
| BAZ2B |  |
| EIF3E |  |
| CWC15 |  |
| TOP1 |  |
| RPL31 |  |
| SUPT6H |  |
| TST |  |
| HNRNPU |  |
| HNRNPUL1 |  |
| HNRNPUL2 |  |
| RPS25 |  |
| MRPL23 |  |
| EIF1AD |  |
| SETX |  |
| MARS2 |  |
| CNOT3 |  |
| MTIF2 |  |
| JAKMIP1 |  |
| FASTKD1 |  |
