# Supplementary material for: Construction and validation of a prognostic model of RNA binding proteins in clear cell renal carcinoma
Source: BMC Nephrol. 2022 May 5;23:172. doi: 10.1186/s12882-022-02801-y (PMC9069774; doi:10.1186/s12882-022-02801-y)
Supplement: Supplementary file 2 — Additional file 2: Supplement Table 2. Results of differential expression analysis of RBPs in the TCGA database ccRCC. [file 12882_2022_2801_MOESM2_ESM.docx]

| **Supplement Table 2:** Results of differential expression analysis of RBPs in the TCGA database ccRCC. | | | | | |
| --- | --- | --- | --- | --- | --- |
| Gene | conMean | treatMean | logFC | pValue | fdr |
| MSI2 | 8.888390976 | 4.394206481 | -1.016319616 | 6.45E-34 | 2.01E-32 |
| LARS2 | 8.680355844 | 3.216923411 | -1.432072597 | 8.26E-36 | 4.29E-34 |
| RBFOX1 | 0.103941624 | 0.02673762 | -1.958830552 | 1.36E-34 | 5.51E-33 |
| ELAVL3 | 0.051439083 | 0.148339487 | 1.527965865 | 1.73E-06 | 2.89E-06 |
| ELAVL4 | 0.01559476 | 0.054606498 | 1.80801127 | 1.23E-20 | 5.20E-20 |
| DDX41 | 12.49377081 | 25.1998453 | 1.012205907 | 2.32E-33 | 5.80E-32 |
| CLASRP | 2.621731909 | 5.931301328 | 1.1778285 | 1.81E-25 | 1.27E-24 |
| TDRD9 | 1.453854836 | 0.646701516 | -1.168711329 | 2.06E-21 | 9.25E-21 |
| ZC3H12D | 0.182171785 | 0.516013914 | 1.502110436 | 1.88E-22 | 9.23E-22 |
| PABPN1L | 0.002199218 | 0.011183637 | 2.346326737 | 2.96E-10 | 6.25E-10 |
| EEF1G | 0.082692821 | 0.204553898 | 1.306647036 | 3.66E-11 | 8.32E-11 |
| RDM1 | 0.051127507 | 0.207088369 | 2.018074938 | 2.85E-24 | 1.68E-23 |
| MRPL33 | 35.64064148 | 16.15507681 | -1.141535694 | 7.52E-40 | 3.51E-37 |
| APOBEC3H | 0.234198372 | 1.628152302 | 2.797432706 | 8.04E-37 | 7.04E-35 |
| APOBEC3F | 1.284951936 | 2.651335436 | 1.045004809 | 4.48E-33 | 1.03E-31 |
| APOBEC3G | 1.203538657 | 5.718305482 | 2.248305213 | 3.53E-36 | 2.36E-34 |
| APOBEC1 | 0.000993601 | 0.011523447 | 3.535761375 | 0.0002614 | 0.000372 |
| PATL2 | 0.096574333 | 0.678263058 | 2.812133197 | 2.58E-37 | 2.78E-35 |
| DAZL | 0.03643725 | 0.007578521 | -2.265425839 | 1.61E-10 | 3.47E-10 |
| FBXO17 | 8.53246279 | 24.87253675 | 1.543519533 | 8.73E-32 | 1.57E-30 |
| TDRD5 | 0.729984598 | 0.093123687 | -2.970645933 | 3.55E-37 | 3.55E-35 |
| POLR2J2 | 0.001700782 | 0.004737387 | 1.477892834 | 0.0141649 | 0.017257 |
| QTRT1 | 5.543731571 | 12.14908645 | 1.131918526 | 1.65E-24 | 1.01E-23 |
| AZGP1 | 59.35239894 | 12.47943961 | -2.249753192 | 1.40E-06 | 2.36E-06 |
| PRDX1 | 311.1103 | 140.7543592 | -1.144246555 | 7.56E-39 | 1.76E-36 |
| RBM44 | 0.040269759 | 0.110357364 | 1.454414147 | 8.39E-22 | 3.89E-21 |
| OASL | 1.132285584 | 3.196449766 | 1.497232542 | 3.25E-23 | 1.70E-22 |
| OAS1 | 5.072833029 | 13.38994637 | 1.400286602 | 6.82E-30 | 8.53E-29 |
| OAS2 | 3.901053414 | 9.63922811 | 1.305053871 | 1.53E-26 | 1.20E-25 |
| RNASE10 | 0.031220407 | 0.15222179 | 2.285613647 | 1.45E-23 | 8.00E-23 |
| NOL3 | 3.73775042 | 38.60298701 | 3.36847034 | 7.07E-40 | 3.51E-37 |
| ADAD1 | 0.000362574 | 0.005552995 | 3.936916904 | 3.98E-06 | 6.51E-06 |
| ADAD2 | 0.061579899 | 0.026074806 | -1.23980297 | 1.29E-08 | 2.48E-08 |
| CLK4 | 1.890534585 | 5.286204466 | 1.483437986 | 5.79E-33 | 1.31E-31 |
| CLK1 | 8.505581456 | 19.28702128 | 1.181148579 | 7.30E-19 | 2.83E-18 |
| CLK2 | 5.159263366 | 10.62006156 | 1.04155513 | 1.41E-23 | 7.79E-23 |
| EIF4A1 | 0.466100754 | 1.876824883 | 2.009580293 | 3.29E-30 | 4.35E-29 |
| IGF2BP2 | 3.012189904 | 1.403915895 | -1.101356218 | 1.63E-22 | 8.09E-22 |
| IGF2BP3 | 0.039654132 | 0.331343531 | 3.062784661 | 1.27E-15 | 3.85E-15 |
| TDRD10 | 0.37950613 | 0.79228923 | 1.061904003 | 5.15E-11 | 1.16E-10 |
| IPO13 | 15.73299779 | 7.421196219 | -1.084069932 | 1.38E-34 | 5.51E-33 |
| MOV10L1 | 0.042883522 | 0.125849744 | 1.553206966 | 3.56E-18 | 1.30E-17 |
| AFF3 | 0.693244881 | 1.723728956 | 1.314095975 | 1.58E-15 | 4.75E-15 |
| AFF2 | 0.113690997 | 0.256548794 | 1.174115225 | 4.30E-15 | 1.25E-14 |
| RBM46 | 0.010581404 | 0.120700196 | 3.511825073 | 1.60E-25 | 1.13E-24 |
| RBM47 | 34.23635536 | 15.53063916 | -1.140411923 | 2.07E-36 | 1.52E-34 |
| KHDC1L | 0.00378118 | 0.02213483 | 2.549409938 | 2.99E-10 | 6.31E-10 |
| RPL10L | 0.010521414 | 0.033538402 | 1.672485392 | 4.40E-06 | 7.18E-06 |
| C2orf15 | 3.021030557 | 0.83730051 | -1.851223367 | 1.86E-37 | 2.37E-35 |
| RNF113B | 0.016884144 | 0.097282901 | 2.526517212 | 3.18E-06 | 5.23E-06 |
| NOP16 | 3.018733589 | 6.314391663 | 1.064700309 | 3.70E-30 | 4.84E-29 |
| U2AF1 | 0.035293174 | 0.106944647 | 1.599403186 | 5.18E-14 | 1.39E-13 |
| U2AF1L4 | 1.381562134 | 2.927851528 | 1.083541949 | 2.00E-17 | 6.90E-17 |
| RNASE9 | 0.000698968 | 0.003866522 | 2.467739152 | 1.42E-05 | 2.22E-05 |
| ZC3HAV1L | 2.086981918 | 4.696620005 | 1.170204772 | 2.80E-22 | 1.35E-21 |
| TDRD6 | 0.078848691 | 0.303681274 | 1.945399241 | 9.84E-30 | 1.17E-28 |
| TDRD15 | 0.009275879 | 0.004069346 | -1.188687001 | 5.02E-13 | 1.27E-12 |
| NXF5 | 0.019811687 | 0.080625451 | 2.024883581 | 3.18E-17 | 1.08E-16 |
| NXF3 | 0.140513202 | 0.065059321 | -1.110878006 | 1.76E-08 | 3.38E-08 |
| PIWIL3 | 0.004371823 | 0.021707789 | 2.311905961 | 1.76E-05 | 2.74E-05 |
| PIWIL4 | 0.378343573 | 0.877849386 | 1.214276498 | 2.05E-25 | 1.43E-24 |
| ACO1 | 27.92906787 | 12.49841582 | -1.16002218 | 3.71E-23 | 1.92E-22 |
| ZCCHC13 | 0.000117437 | 0.003646004 | 4.956355864 | 0.022541 | 0.026945 |
| DDX39B | 3.858249838 | 11.62764142 | 1.591540015 | 4.33E-21 | 1.91E-20 |
| RNASE2 | 0.412047777 | 2.073613679 | 2.331263608 | 2.22E-25 | 1.53E-24 |
| RNASE3 | 0.035154687 | 0.191674376 | 2.446868518 | 5.83E-26 | 4.30E-25 |
| RNASE6 | 7.14376972 | 20.39079444 | 1.513160504 | 2.71E-24 | 1.61E-23 |
| RNASE8 | 0.007061819 | 0.014837307 | 1.071117609 | 0.00261 | 0.003401 |
| ANG | 5.620423474 | 17.34913107 | 1.626112667 | 4.10E-24 | 2.38E-23 |
| RBMY1E | 0.002425935 | 0.000806708 | -1.588421973 | 0.0056716 | 0.007166 |
| AUH | 24.36000678 | 10.73944759 | -1.181594748 | 8.30E-33 | 1.79E-31 |
| PIH1D3 | 0.022544794 | 0.010269033 | -1.134493968 | 4.81E-08 | 8.97E-08 |
| CSDC2 | 9.763054231 | 3.644401876 | -1.421650488 | 8.02E-16 | 2.47E-15 |
| DDX25 | 0.345455723 | 0.059504975 | -2.537418609 | 1.70E-37 | 2.37E-35 |
| EXOSC5 | 5.922851768 | 13.96928943 | 1.237894752 | 1.13E-33 | 3.10E-32 |
| EZH2 | 0.53786403 | 1.827139657 | 1.764273493 | 5.43E-38 | 1.03E-35 |
| KHDRBS2 | 0.273243408 | 0.070110584 | -1.962482543 | 1.71E-17 | 5.97E-17 |
| PPARGC1A | 14.64665297 | 4.872718645 | -1.587772192 | 1.67E-34 | 6.32E-33 |
| C9orf129 | 0.017420851 | 0.042471566 | 1.285682165 | 0.0045621 | 0.005832 |
| LUC7L | 2.763314778 | 5.626357845 | 1.025801399 | 3.43E-13 | 8.82E-13 |
| TDRD1 | 0.531510231 | 0.133560976 | -1.992598926 | 5.49E-36 | 2.96E-34 |
| ENOX1 | 2.276309803 | 0.590863619 | -1.945799843 | 5.45E-29 | 5.78E-28 |
| TRMT1 | 4.158438538 | 8.981093705 | 1.110849236 | 5.86E-38 | 1.03E-35 |
| DDX47 | 0.154162183 | 0.454724348 | 1.560543345 | 8.57E-29 | 8.83E-28 |
| SAMHD1 | 9.732743202 | 26.15865593 | 1.426370019 | 3.51E-25 | 2.33E-24 |
| RALYL | 3.867331076 | 0.071547186 | -5.756299433 | 1.55E-40 | 2.17E-37 |
| NPM2 | 1.771358075 | 0.84778522 | -1.063085158 | 8.96E-24 | 5.00E-23 |
| SNRPN | 53.90382823 | 20.67973678 | -1.382169914 | 1.09E-39 | 3.82E-37 |
| DARS | 24.7006539 | 51.79097076 | 1.068151366 | 4.02E-32 | 7.88E-31 |
| RBM11 | 1.10220449 | 0.195898645 | -2.492212588 | 4.58E-36 | 2.57E-34 |
| POLR2F | 0.016320496 | 0.050374969 | 1.626022099 | 1.47E-28 | 1.47E-27 |
| SNRNP70 | 14.88057675 | 29.78988868 | 1.001392288 | 3.09E-17 | 1.05E-16 |
| TLR7 | 0.710926892 | 2.840118696 | 1.998178111 | 2.59E-24 | 1.55E-23 |
| TLR8 | 0.426893513 | 1.757216481 | 2.041343792 | 3.68E-23 | 1.91E-22 |
| TLR3 | 5.587689332 | 16.19081368 | 1.534851775 | 4.97E-25 | 3.26E-24 |
| DQX1 | 0.011786685 | 0.081223541 | 2.784739862 | 2.72E-13 | 7.05E-13 |
| CELF3 | 0.051929054 | 0.015634036 | -1.731851664 | 0.0001276 | 0.000186 |
| CELF4 | 0.120262166 | 0.255889866 | 1.089340167 | 0.0034633 | 0.004468 |
| CELF6 | 0.007484791 | 0.018727617 | 1.323133371 | 8.32E-08 | 1.52E-07 |
| DDX53 | 0.000748186 | 0.008208041 | 3.455568246 | 0.000175 | 0.000253 |
| CPEB3 | 3.338157511 | 1.276648898 | -1.386690218 | 2.40E-35 | 1.16E-33 |
| PABPC1L | 0.969098661 | 4.32157791 | 2.156842716 | 9.55E-18 | 3.41E-17 |
| PABPC4L | 0.94133138 | 2.127258095 | 1.176220488 | 2.50E-15 | 7.42E-15 |
| MEX3B | 0.343184182 | 0.752661327 | 1.133017788 | 1.93E-17 | 6.65E-17 |
| TERT | 0.003895539 | 0.121695052 | 4.965303758 | 2.45E-24 | 1.47E-23 |
| ISG20 | 0.453110191 | 2.176056497 | 2.263782169 | 1.06E-36 | 8.76E-35 |
| AEN | 2.501645011 | 6.41688275 | 1.35899554 | 2.21E-37 | 2.58E-35 |
| RPS19 | 84.98449456 | 177.2520667 | 1.060530898 | 4.19E-36 | 2.45E-34 |
| YBX2 | 0.312035084 | 0.736479095 | 1.238936328 | 0.0186082 | 0.022436 |
| YBX3 | 12.53423366 | 34.90252847 | 1.477457762 | 4.14E-36 | 2.45E-34 |
| THOC6 | 6.893162049 | 14.23938896 | 1.046649402 | 3.54E-33 | 8.40E-32 |
| RPL22L1 | 3.673889284 | 9.109939966 | 1.310133396 | 1.27E-27 | 1.12E-26 |
| ESRP1 | 7.637564547 | 0.857782868 | -3.15442826 | 2.42E-36 | 1.70E-34 |
| ESRP2 | 6.583540294 | 2.254518637 | -1.546044165 | 8.72E-36 | 4.36E-34 |
| ARL6IP4 | 0.306684377 | 0.891106604 | 1.538843357 | 6.89E-23 | 3.50E-22 |
| RNASET2 | 3.427779116 | 33.35102921 | 3.282385233 | 3.95E-36 | 2.45E-34 |
| RPL36A | 7.612887806 | 17.00518758 | 1.159459199 | 9.23E-32 | 1.64E-30 |
| EXO1 | 0.163327613 | 0.521250814 | 1.674209009 | 2.86E-31 | 4.67E-30 |
| NANOS1 | 1.456769015 | 0.481271274 | -1.597849923 | 1.33E-22 | 6.62E-22 |
| NANOS2 | 0.002461127 | 0.027271486 | 3.470002362 | 5.51E-07 | 9.55E-07 |
| NOVA2 | 0.943721506 | 1.979152007 | 1.068449336 | 1.88E-17 | 6.50E-17 |
| MRPS6 | 38.75559088 | 10.84311562 | -1.837625094 | 9.78E-23 | 4.89E-22 |
| CNP | 28.49764538 | 11.07715601 | -1.363255196 | 1.23E-36 | 9.57E-35 |
| TST | 47.13613159 | 18.65426986 | -1.337327469 | 4.76E-16 | 1.50E-15 |
| JAKMIP1 | 0.050439067 | 0.55852221 | 3.469001156 | 8.97E-35 | 3.93E-33 |
