# Supplementary material for: Construction and validation of a prognostic model of RNA binding proteins in clear cell renal carcinoma
Source: BMC Nephrol. 2022 May 5;23:172. doi: 10.1186/s12882-022-02801-y (PMC9069774; doi:10.1186/s12882-022-02801-y)
Supplement: Supplementary file 3 — Additional file 3: Supplement Table 3: Number of nodes in the PPI network. [file 12882_2022_2801_MOESM3_ESM.docx]

**Supplement Table 3:** Number of nodes in the PPI network.

| Gene | Count |
| --- | --- |
| DAZL | 12 |
| PIWIL4 | 12 |
| DDX39B | 11 |
| TDRD1 | 11 |
| TDRD5 | 11 |
| EIF4A1 | 10 |
| POLR2F | 10 |
| TDRD9 | 10 |
| ELAVL4 | 9 |
| OASL | 9 |
| RBFOX1 | 9 |
| RPL10L | 9 |
| SNRNP70 | 9 |
| TDRD6 | 9 |
| EEF1G | 8 |
| ELAVL3 | 8 |
| TLR3 | 8 |
| TLR7 | 8 |
| ESRP1 | 7 |
| MOV10L1 | 7 |
| NOP16 | 7 |
| OAS1 | 7 |
| RPL22L1 | 7 |
| RPL36A | 7 |
| RPS19 | 7 |
| SAMHD1 | 7 |
| SNRPN | 7 |
| TDRD10 | 7 |
| YBX2 | 7 |
| DDX25 | 6 |
| DDX47 | 6 |
| ESRP2 | 6 |
| EXOSC5 | 6 |
| ISG20 | 6 |
| NANOS2 | 6 |
| OAS2 | 6 |
| U2AF1L4 | 6 |
| EZH2 | 5 |
| NOVA2 | 5 |
| PABPC1L | 5 |
| PIWIL3 | 5 |
| RNASE2 | 5 |
| TLR8 | 5 |
| ANG | 4 |
| APOBEC3G | 4 |
| CELF3 | 4 |
| CELF4 | 4 |
| CLASRP | 4 |
| CLK1 | 4 |
| DARS | 4 |
| DDX41 | 4 |
| NANOS1 | 4 |
| RNASET2 | 4 |
| TDRD15 | 4 |
| TRMT1 | 4 |
| ADAD1 | 3 |
| CLK2 | 3 |
| DQX1 | 3 |
| EXO1 | 3 |
| KHDRBS2 | 3 |
| LUC7L | 3 |
| MRPS6 | 3 |
| NXF5 | 3 |
| RBM47 | 3 |
| RNASE3 | 3 |
| TERT | 3 |
| THOC6 | 3 |
| APOBEC1 | 2 |
| APOBEC3F | 2 |
| CLK4 | 2 |
| MRPL33 | 2 |
| RBM44 | 2 |
| ACO1 | 1 |
| AEN | 1 |
| AFF3 | 1 |
| APOBEC3H | 1 |
| CELF6 | 1 |
| DDX53 | 1 |
| ENOX1 | 1 |
| IPO13 | 1 |
| LARS2 | 1 |
| MEX3B | 1 |
| MSI2 | 1 |
| NPM2 | 1 |
| NXF3 | 1 |
| PABPN1L | 1 |
| PATL2 | 1 |
| PPARGC1A | 1 |
| PRDX1 | 1 |
| QTRT1 | 1 |
| RALYL | 1 |
| RNASE10 | 1 |
| RNASE6 | 1 |
| RNASE8 | 1 |
| RNF113B | 1 |
| TST | 1 |
| YBX3 | 1 |
| ZC3H12D | 1 |
| ZC3HAV1L | 1 |
| ZCCHC13 | 1 |
