# Supplementary material for: Construction and validation of a prognostic model of RNA binding proteins in clear cell renal carcinoma
Source: BMC Nephrol. 2022 May 5;23:172. doi: 10.1186/s12882-022-02801-y (PMC9069774; doi:10.1186/s12882-022-02801-y)
Supplement: Supplementary file 4 — Additional file 4: Supplementary Figure 1. Survival analysis of 17 survival-related RBPs in ccRCC. [file 12882_2022_2801_MOESM4_ESM.docx]

**
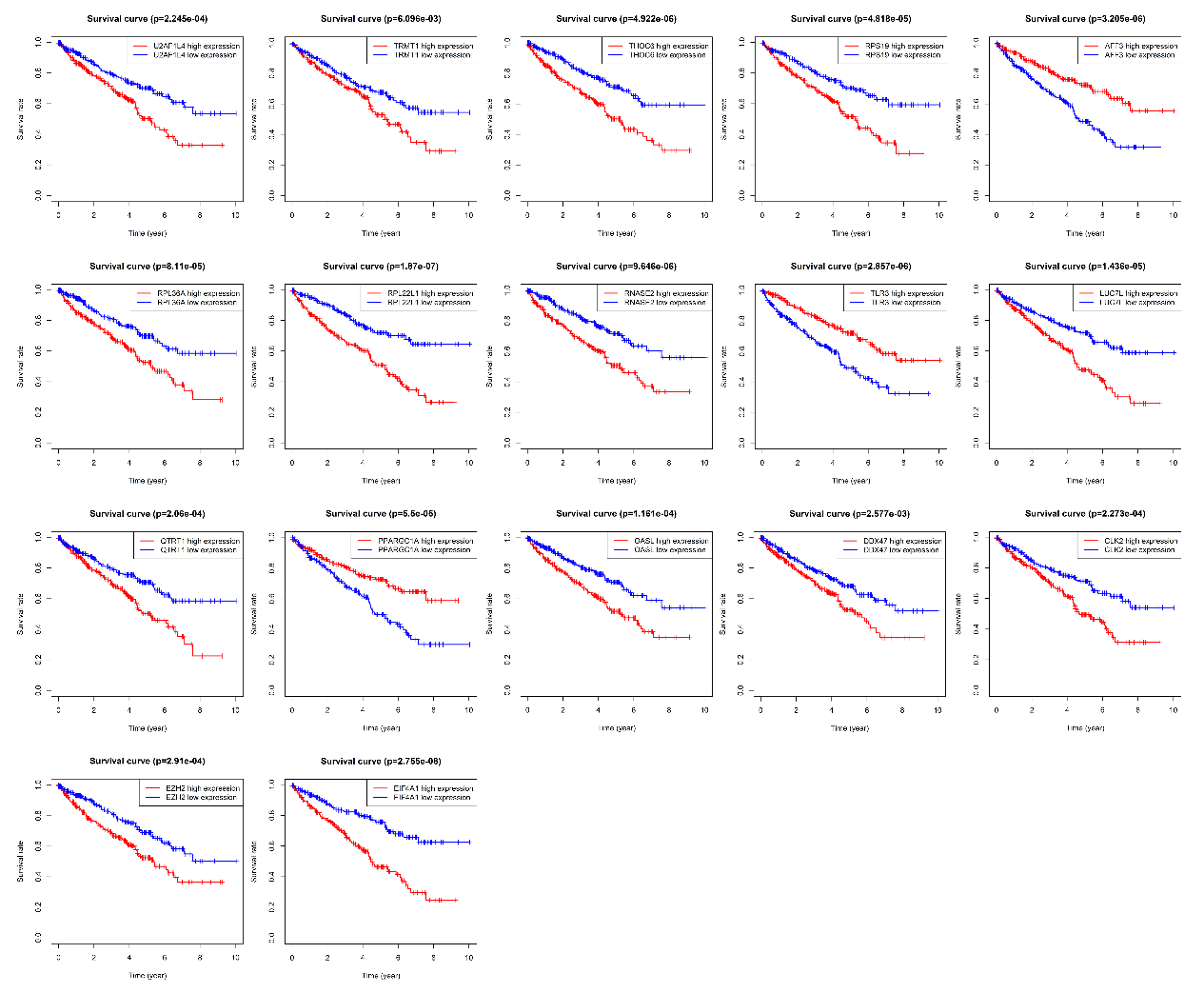
**

**Supplementary Figure 1:** Survival analysis of 17 survival-related RBPs in ccRCC. Patients were divided into high and low expression groups, using the median value of each gene expressed as a cut-off. The survival status of the two groups for each gene was compared using K-M survival analysis.

**17 survival-related genes:** PARGC1A, LUC7L, CLK2, THOC6, RPS19, U2AF1L4, TRMT1, QTRT1, RPL36A, RPL22L1, AFF3, O ASL,TLR3, DDX47, EZH2, EIF4A1, RNASE2.
