# Supplementary material for: Construction and validation of a prognostic model of RNA binding proteins in clear cell renal carcinoma
Source: BMC Nephrol. 2022 May 5;23:172. doi: 10.1186/s12882-022-02801-y (PMC9069774; doi:10.1186/s12882-022-02801-y)
Supplement: Supplementary file 5 — Additional file 5. RNA extraction from tissues. [file 12882_2022_2801_MOESM5_ESM.docx]

**RNA extraction from tissues**

1. The tissue is ground in liquid nitrogen, Trizol reagent (Vazyme, China) is added to disintegrate the tissue (1ml Trizol per 50mg of tissue), and grind with a grinder.
2. The tissue was centrifuged at 10,000 x g for 10 min at 2-8°C and the supernatant was retained.
3. Dispense the supernatant into an EP tube and add chloroform (0.2 ml chloroform per 1 ml Trizol), shake vigorously for 15s and leave at room temperature for 5min.
4. The EP tubes were placed in a centrifuge for 15 minutes (2-8°C, 10,000 x g) and the supernatant was extracted. Transfer the supernatant to a new EP tube with an equal volume of isopropanol and leave at room temperature for 10min.
5. The EP tubes were centrifuged at 2-8°C for 10 min at 10000 x g. A gelatinous white precipitate (RNA precipitate) appeared on the side and bottom of the tubes, and the supernatant was discarded for the next step.
6. Wash the RNA precipitate with 75℅ pre-cooled ethanol. Add at least 1ml of 75℅ ethanol for every 1ml of Trizol used. Centrifuge at 2-8°C for 5min at 7500×g and discard the supernatant.
7. The EP tubes were air-dried on an ultra-clean bench for about 5-10 min. 25-200 μl of RNase-free water was added to the EP tubes to dissolve the RNA. RNA concentration was measured and Reverse transcription‑quantitative PCR analysis was performed.
